# Supplementary material for: AI-assisted isolation of bioactive Dipyrimicins from Amycolatopsis azurea and identification of its corresponding dip biosynthetic gene cluster
Source: bioRxiv. 2025 Mar 21:2025.03.21.644653. Preprint. [Version 1] doi: 10.1101/2025.03.21.644653 (PMC11957118; doi:10.1101/2025.03.21.644653)
Supplement: Supplement 1 [file media-1.pdf]

Supporting Information for

AI-assisted isolation of bioactive Dipyrimicins from *Amycolatopsis azurea* and identification of its corresponding *dip* biosynthetic gene cluster

Christine Mae F. Ancajas,[*1] Isra E. Shuster,[*1,2] Allison S. Walker[1,2,3]

1. Department of Chemistry, Vanderbilt University, 1234 Stevenson Center Lane, Nashville, TN 37240, United States
2. Department of Biological Sciences, Vanderbilt University, VU Station B, Box 35-1634, Nashville, TN 37235, United States
3. Department of Pathology, Microbiology, and Immunology, 1211 Medical Center Drive, Vanderbilt University Medical Center, Nashville, TN 37232, United States

* authors contributed equally
address correspondence to: Allison Walker (allison.s.walker@vanderbilt.edu)

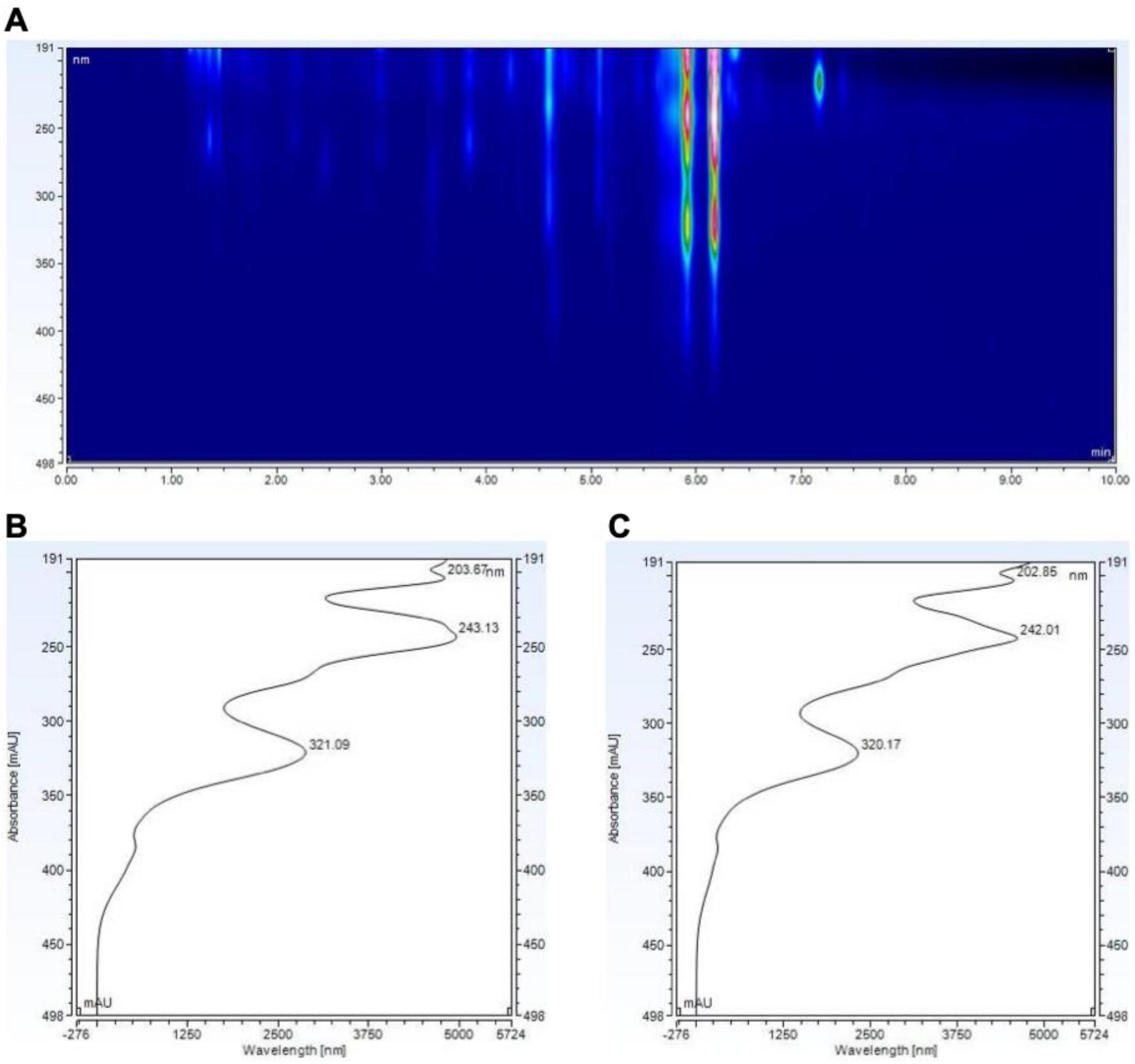

Figure S1. (A) HPLC chromatogram and UV-Vis spectrum of *A. azurea* DSM 43854 culture extract showing typical UV-Vis spectrum for 2,2'-bipyridines (**B**) Dipyrimicin A (compound **1,** λ = 204nm, 243nm, 268nm, and 321nm, retention time (RT) at ~5.8 min) and (**C**) Dipyrimicin B (compound **2,** λ = 203nm, 242nm, 268nm, and 320nm, RT at ~6.2 min).

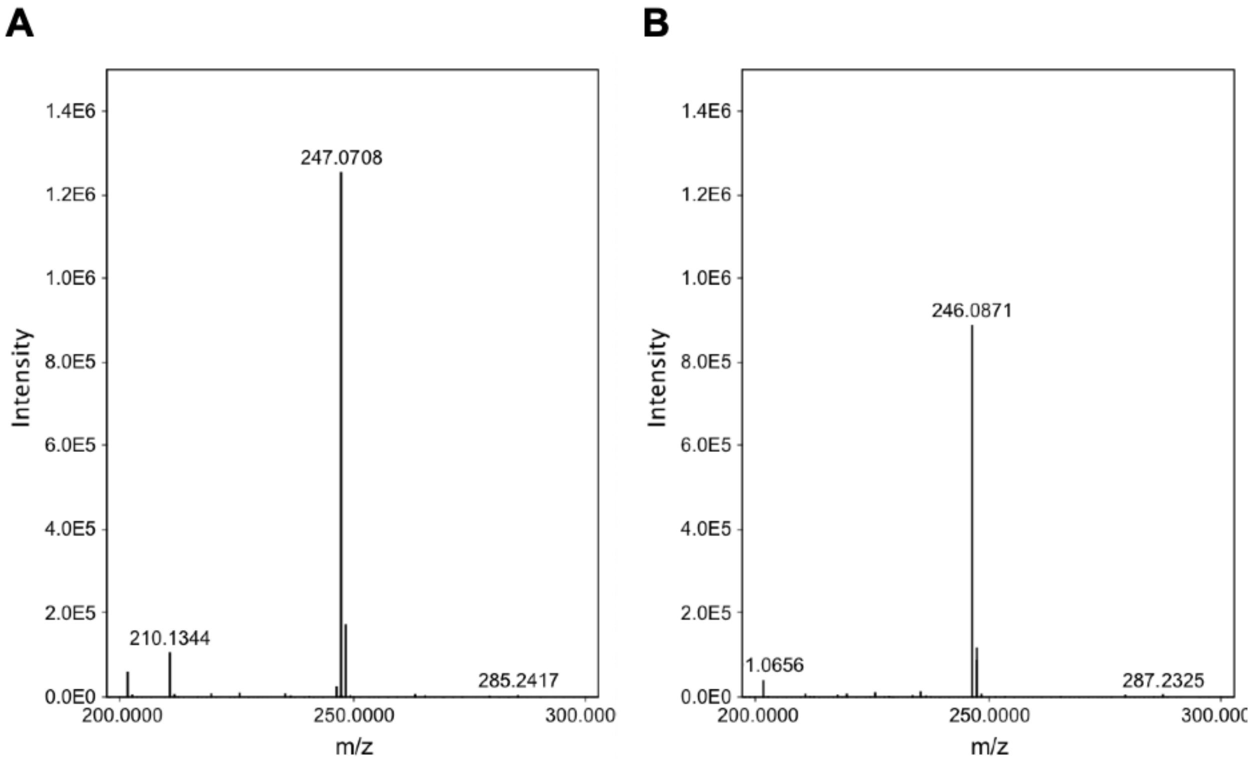

Figure S2. HR-ESI-MS spectrum of **(A)** Dipyrimicin A (compound **1,** m/z $[M+H]^+$ obsvd. 247.0708, calcd. for $C_{12}H_{10}N_2O_4$, 247.0719) and (**B**) Dipyrimicin B (compound **2,** m/z $[M+H]^+$ obsvd. 246.0871, calcd. for $C_{12}H_{11}N_3O_3$, 246.0879)

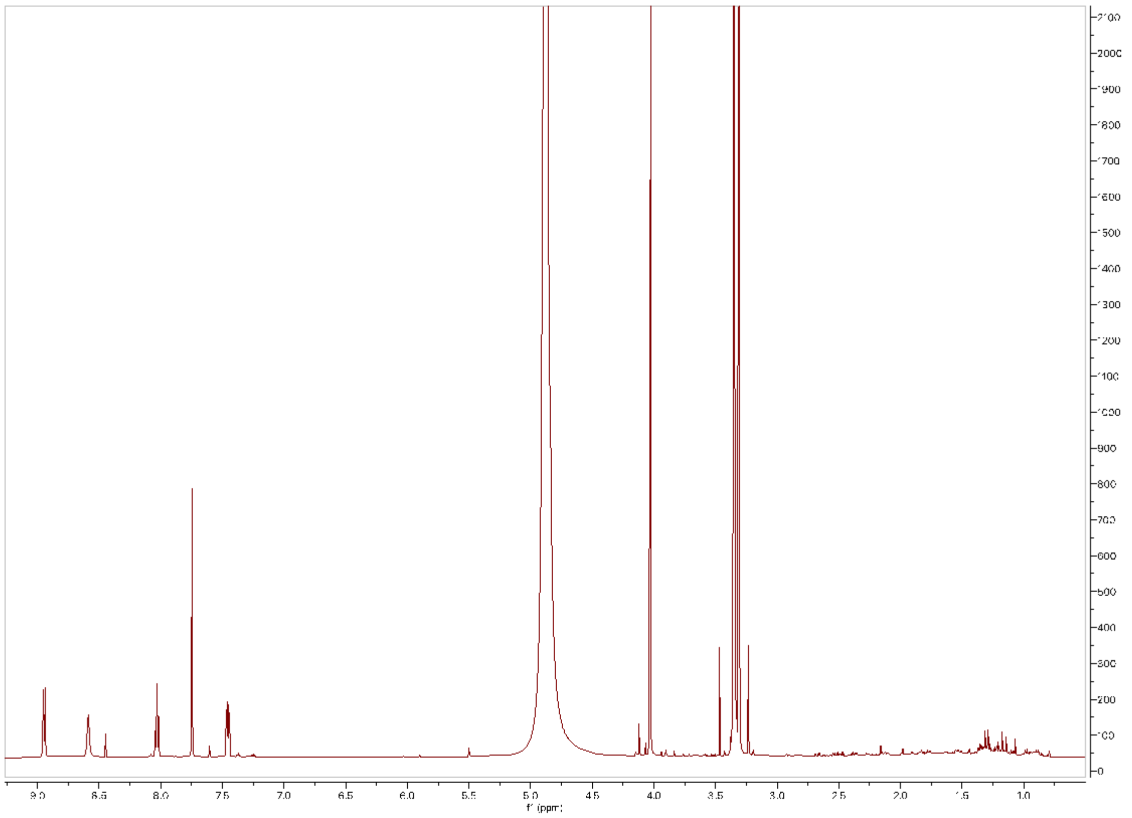

Figure S3. ^{1}H NMR (600 MHz) spectrum of Dipyrimicin A (**1**) in CD_3OD.

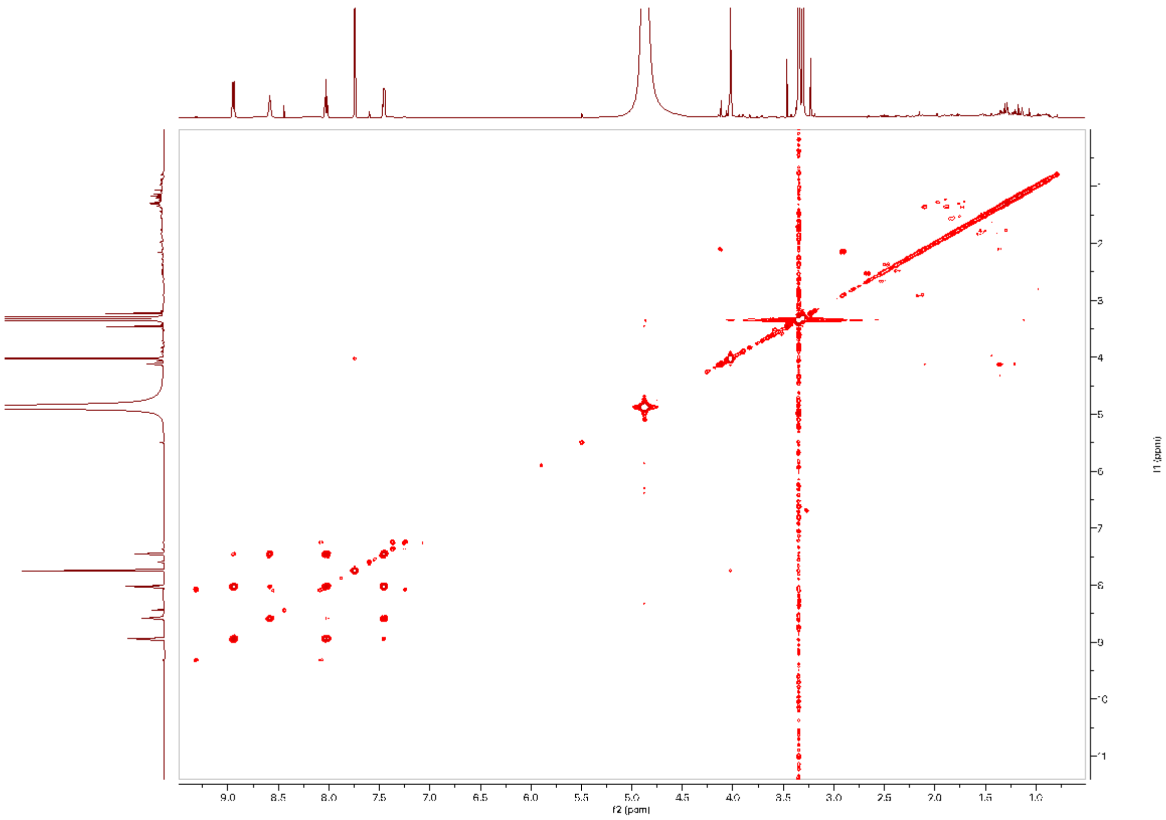

Figure S4. ^{1}H-^{1}H COSY NMR (600 MHz) spectrum of Dipyrimicin A (**1**) in CD_3OD.

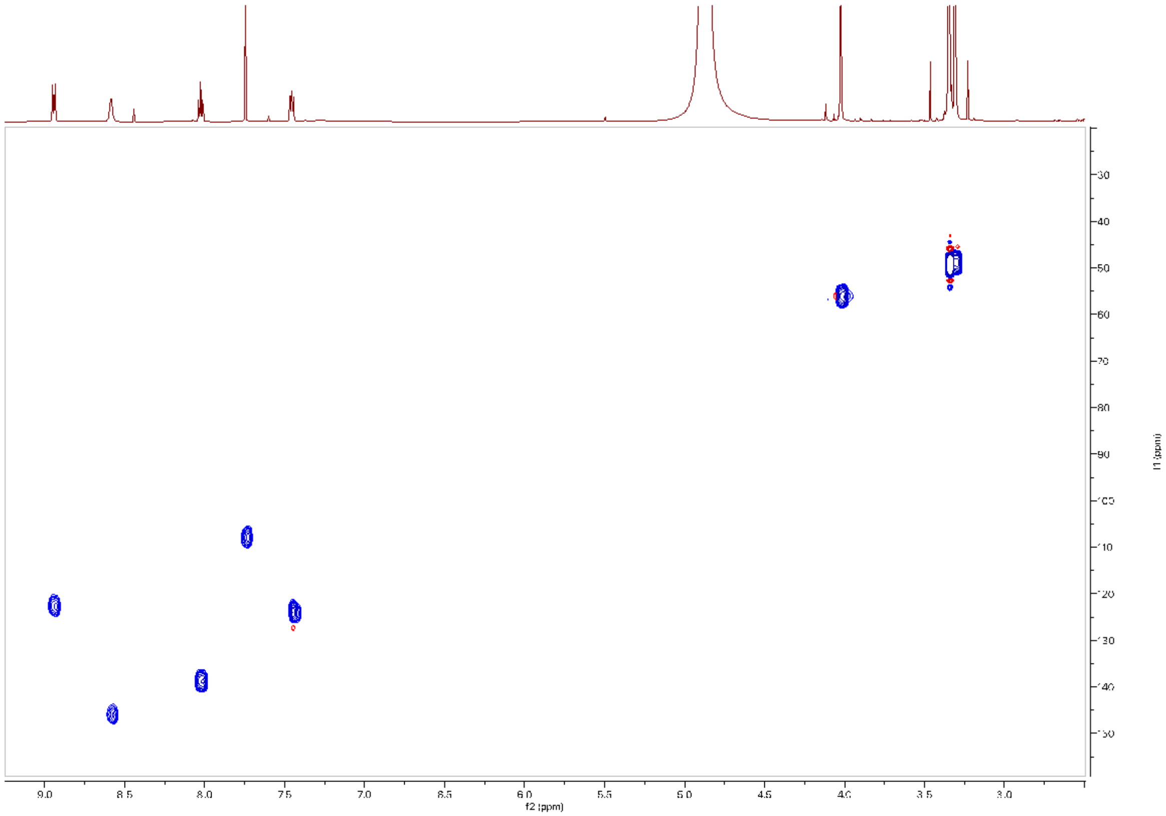

Figure S5. ^{1}H-^{13}C HSQC NMR (600 MHz) spectrum of Dipyrimicin A (**1**) in CD_3OD.

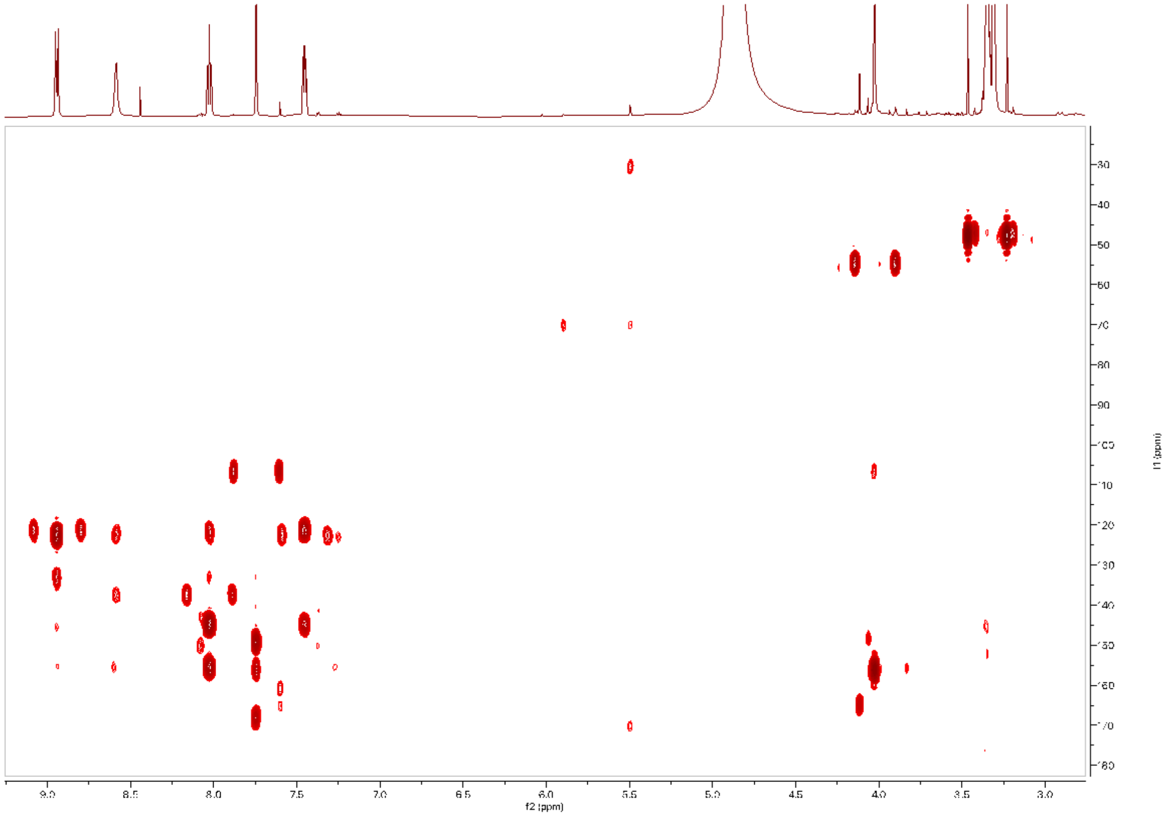

Figure S6. ^{1}H-^{13}C HMBC NMR (600 MHz) spectrum of Dipyrimicin A (**1**) in CD_3OD.

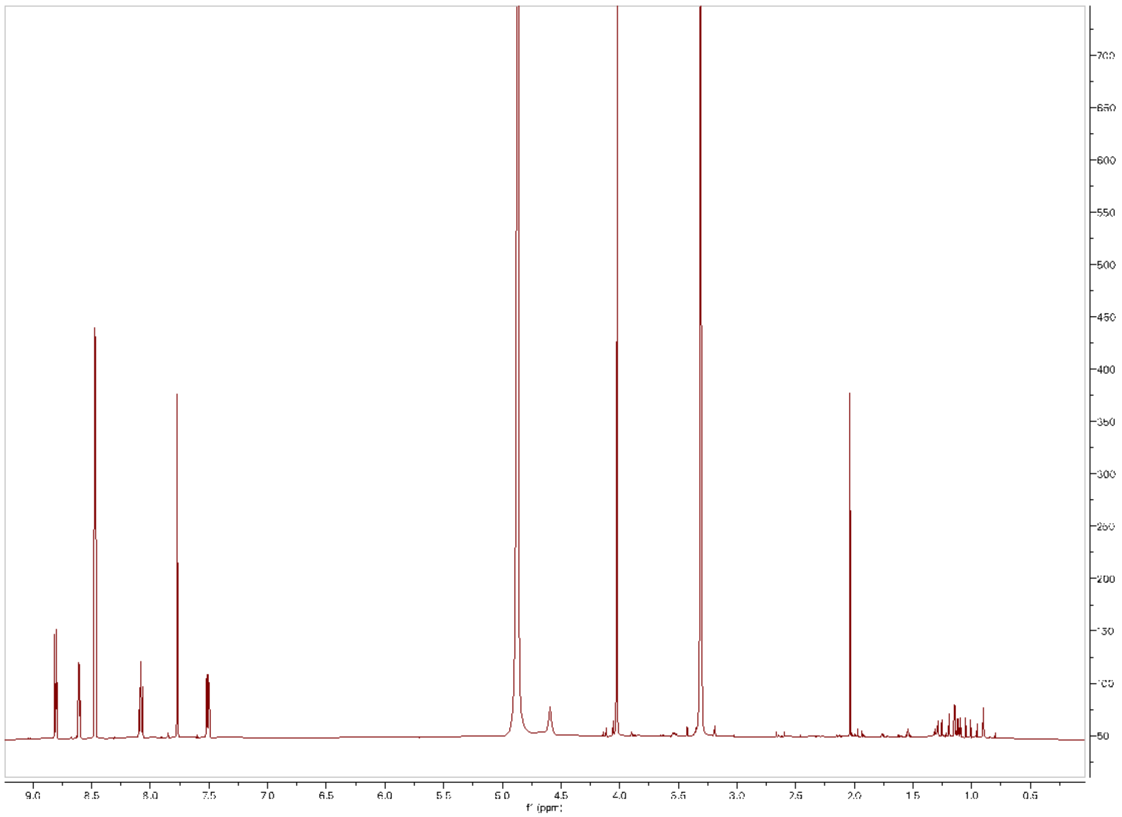

Figure S7. ^{1}H NMR (600 MHz) spectrum of Dipyrimicin B (**2**) in CD_3OD.

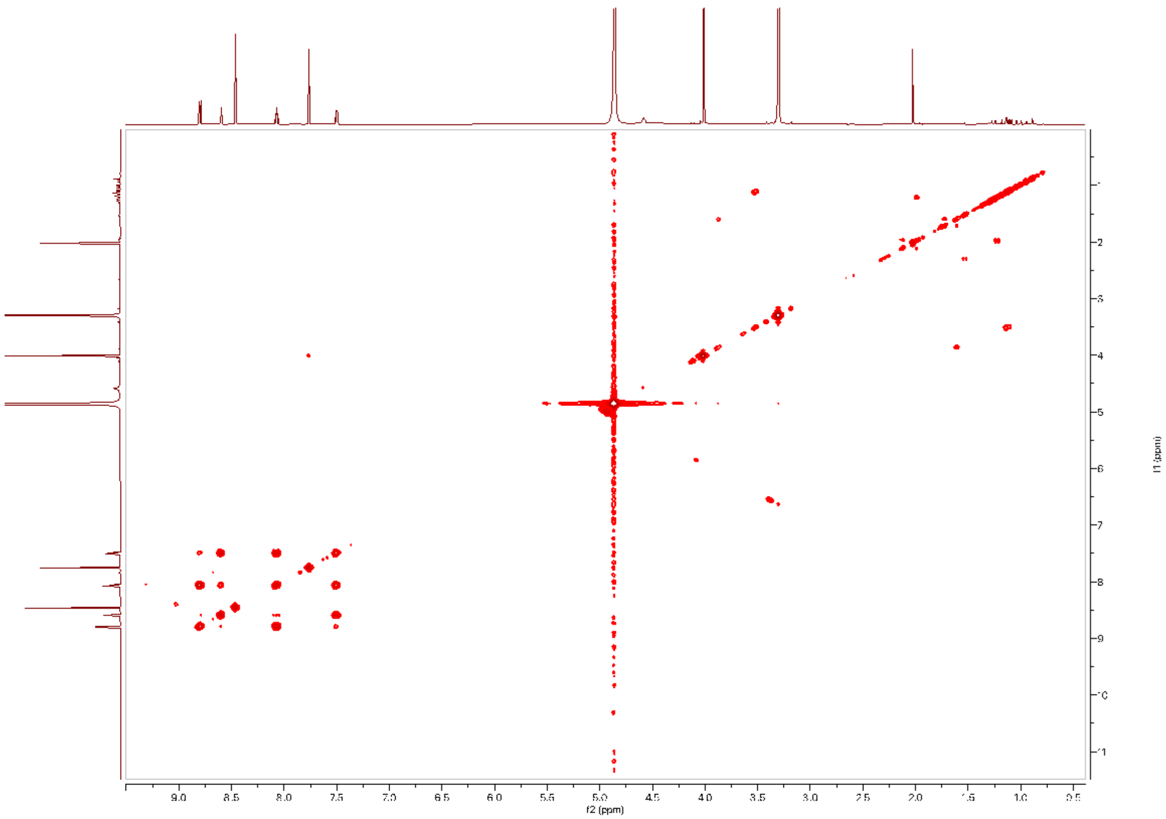

Figure S8. ^{1}H-^{1}H COSY NMR (600 MHz) spectrum of Dipyrimicin B (**2**) in CD_3OD.

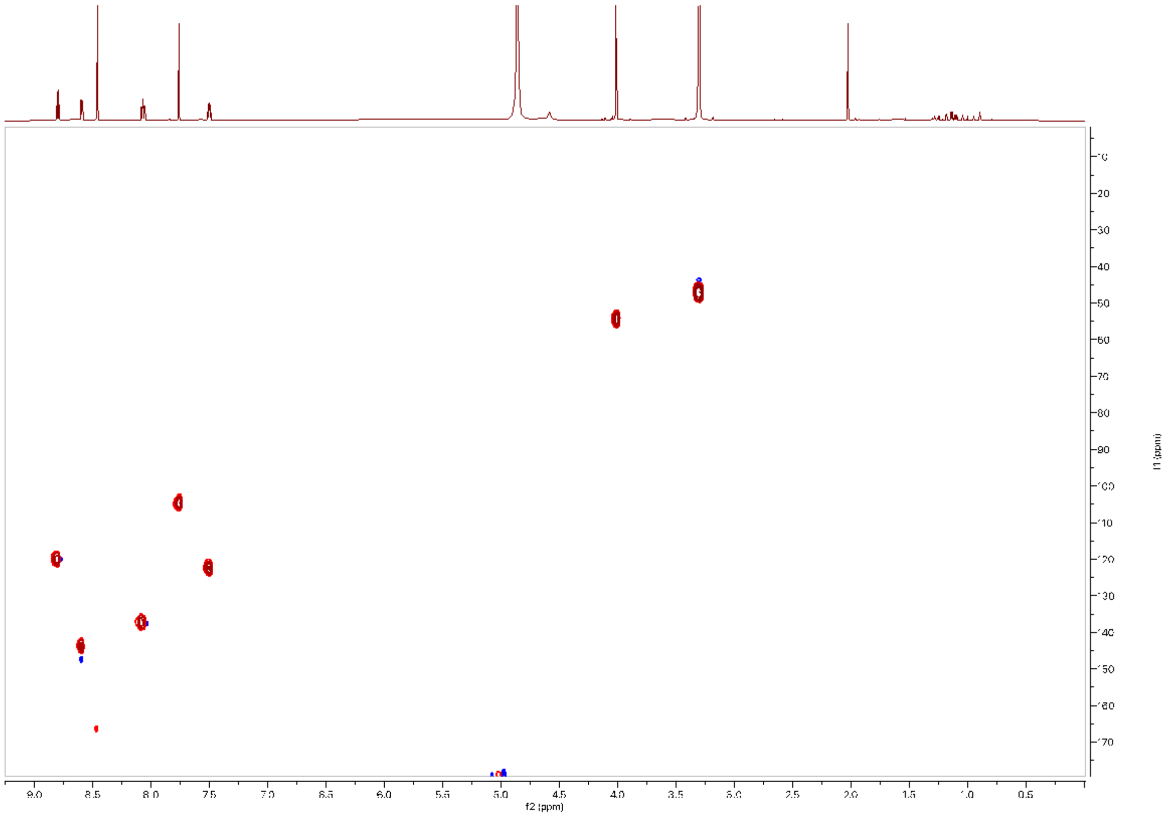

Figure S9. ^{1}H-^{13}C HSQC NMR (600 MHz) spectrum of Dipyrimicin B (**2**) in CD_3OD.

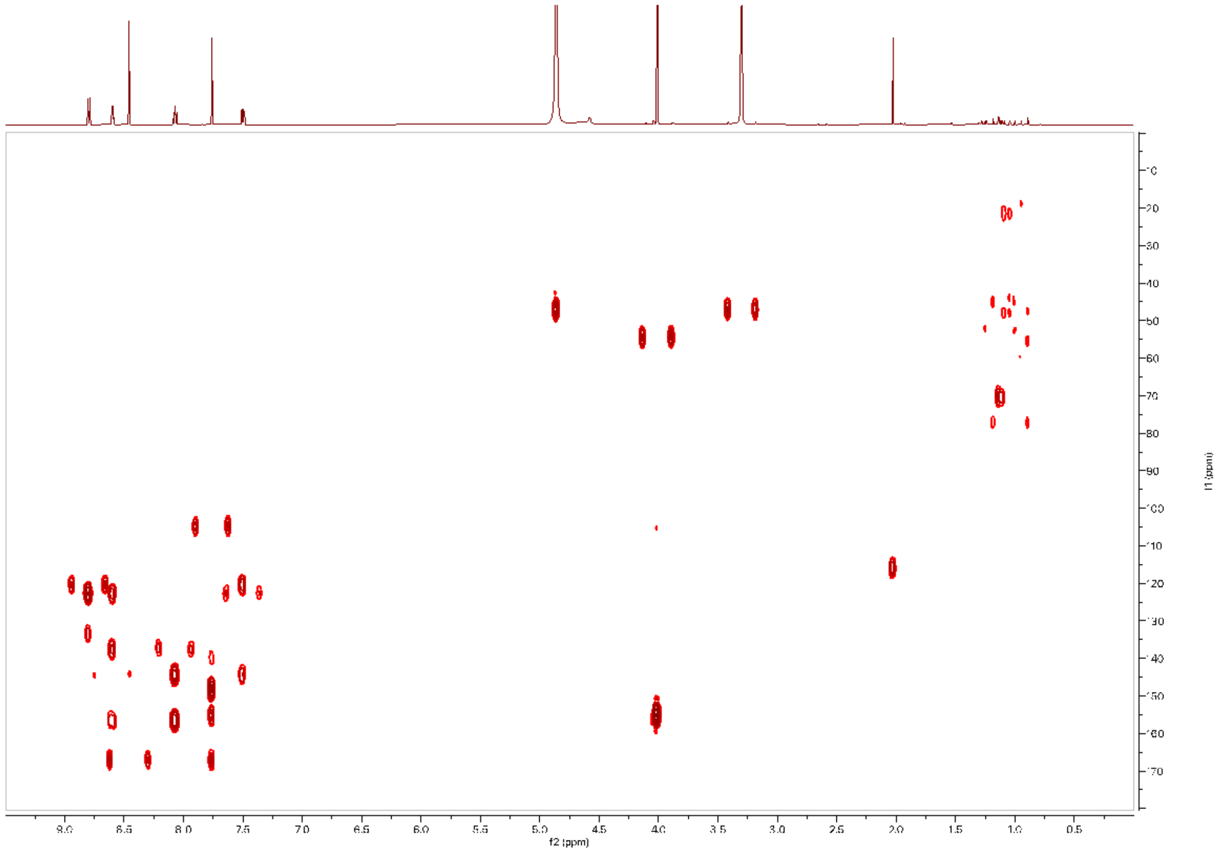

Figure S10. ^{1}H-^{13}C HMBC NMR (600 MHz) spectrum of Dipyrimicin B (**2**) in CD_3OD.

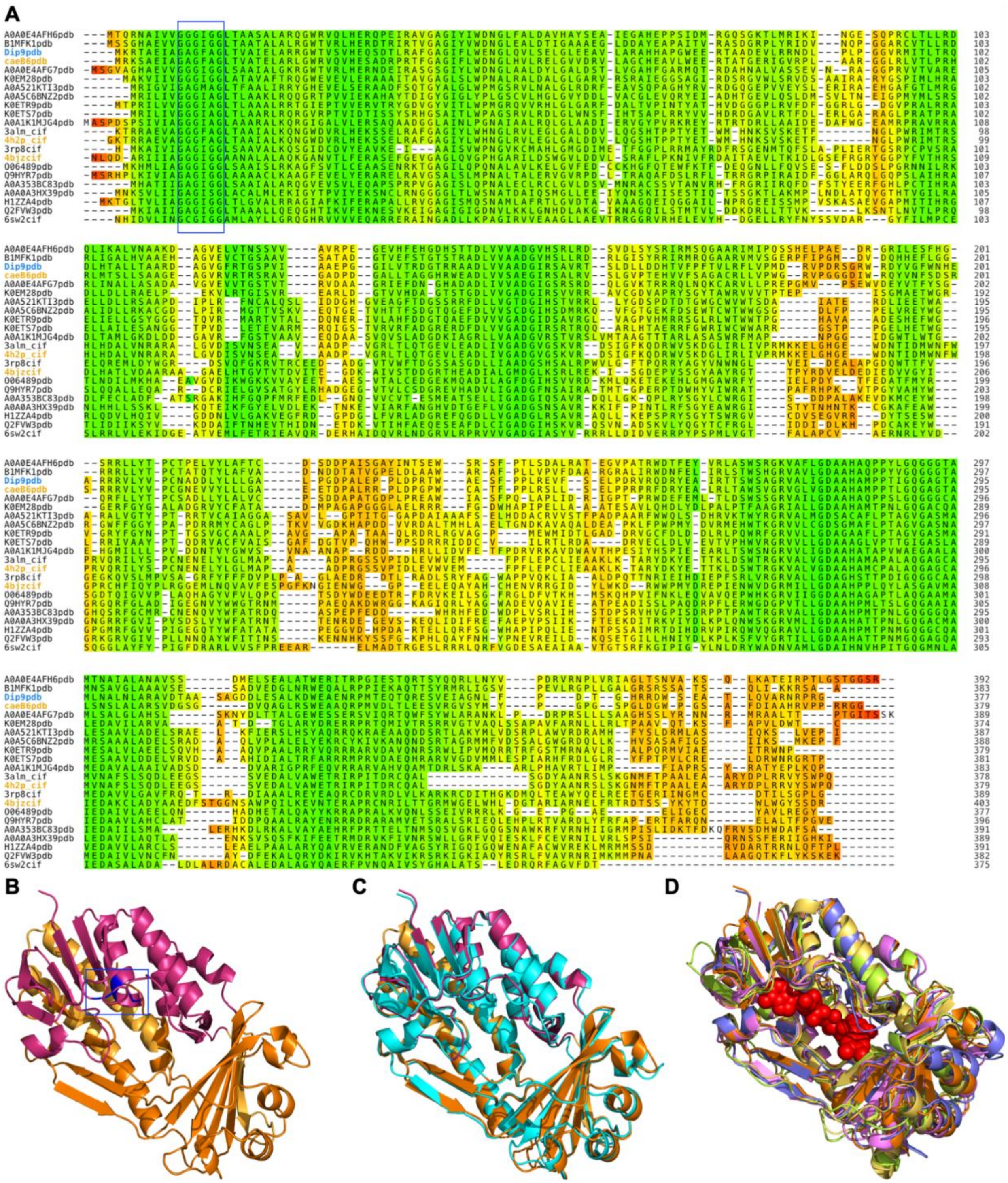

Figure S11. Multiple structural alignment (MSTA) of Dip9 and related hydroxylases. A) FoldMason MSTA of the Dip9 structure along with the top 5 FoldSeek hits from different databases (**Table S4**). Residues are shaded by the LDDT score of their alignment column (color scheme: red 0%, green 100%). Highlighted in the blue box is the conserved "GxGxxG" motif found among flavin-containing oxygenases.[1] B) The AlphaFold-predicted structure of Dip9. Highlighted in pink and dark orange are the putative FAD-binding and substrate-binding domains, respectively. The glycine-rich motif is depicted in blue within the box. C) Superimposition of the

predicted structures of Dip9 and CaeB6 (cyan). D) Superimposition of Dip9 and the top FoldSeek hits from different databases (**Table S4**) and the representative FAD is depicted in red spheres (general location of FAD in PDBs 4H2P and 4BJZ), highlighting that the related hydroxylases have similar sites for FAD- and substrate-binding.

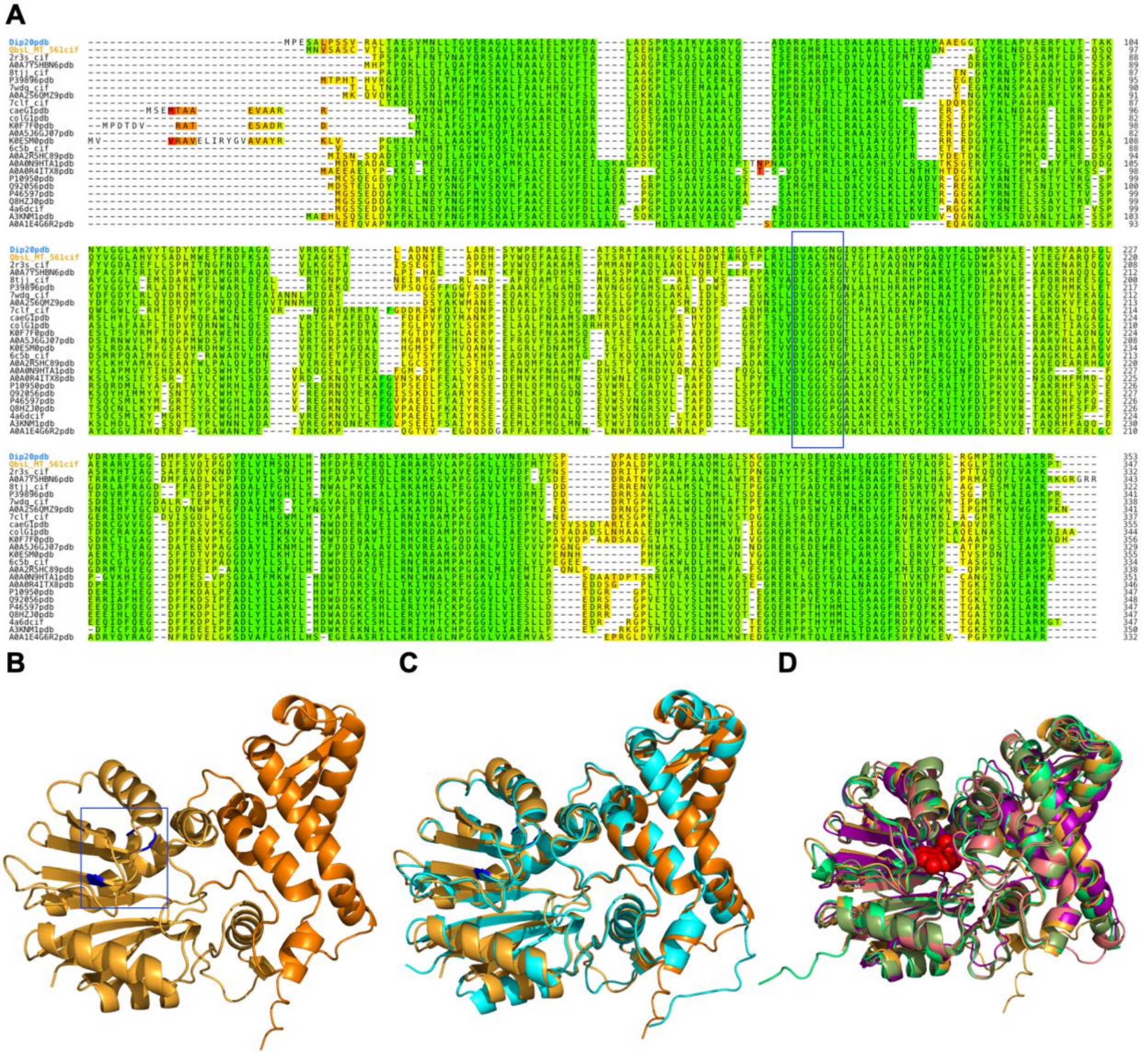

Figure S12. Multiple structural alignment (MSTA) of Dip20 and related methyltransferases. A) FoldMason MSTA of the Dip20 structure along with the top 5 FoldSeek hits from different databases (**Table S5**). Residues are shaded by the LDDT score of their alignment column (color scheme: red 0%, green 100%). Highlighted in the blue box is the “DxGxGxG” or “GxG” fingerprint, a SAM-binding motif found among class I methyltransferases. B) The AlphaFold-predicted structure of Dip20. The C-terminal domain (light orange) contains the pocket for SAM- and substrate-binding while the N-terminal domain is for dimerization.The glycine-rich motif is depicted in blue within the box. C) Superimposition of the predicted structures of Dip20 and the C-terminal domain of QbsL (cyan). D) Superimposition of Dip20 and the top FoldSeek hits from

different databases (**Table S5**). The representative SAM molecule is depicted in red spheres (from 2R3S PDB).

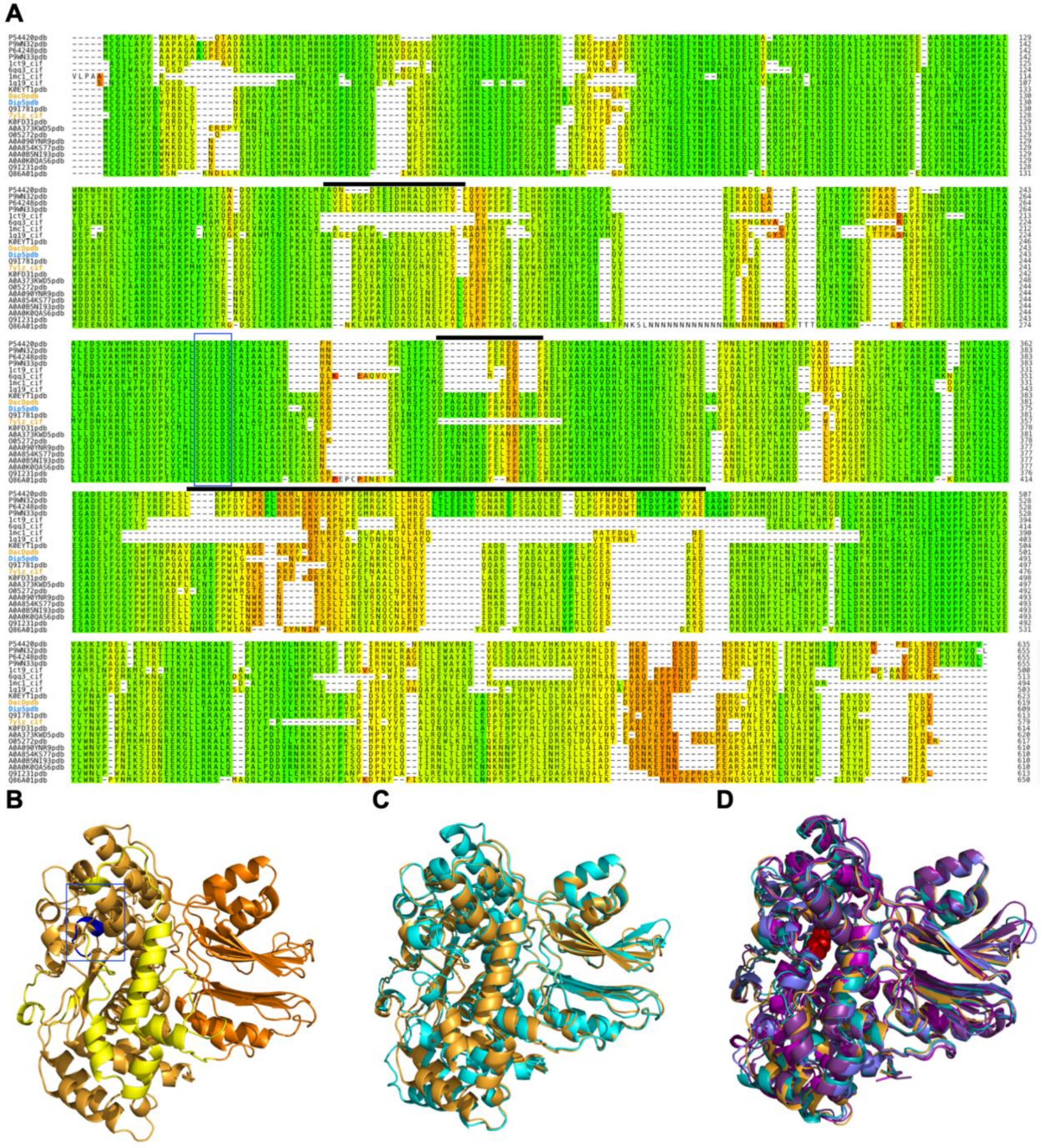

Figure S13. Multiple structural alignment (MSTA) of Dip5 and related amidotransferases. A) FoldMason MSTA of the Dip5 structure along with the top 5 FoldSeek hits from different databases (**Table S6**). Residues are shaded by the LDDT score of their alignment column (color scheme: red 0%, green 100%). Highlighted in the blue box is the conserved “SGGLDS” motif among amidotransferases. Dip5 and several other related proteins reveal inserted regions (black bars) that are absent when compared to the glutamine-dependent asparagine synthetase, AsnB,

from *Escherichia coli* (PDB 1CT9). B) The AlphaFold-predicted structure of Dip5. Highlighted in light and dark orange are the C-terminal synthetase and N-terminal glutaminase domains, respectively. Residues in yellow are the inserted regions not found in AsnB. The conserved motif involved in ATP-binding is depicted in blue within the box. C) Superimposition of the predicted structures of Dip5 and DacD. D) Superimposition of Dip5 and the top FoldSeek hits from different databases (**Table S6**). The representative ATP molecule is depicted in red spheres (from 1CT9 PDB).

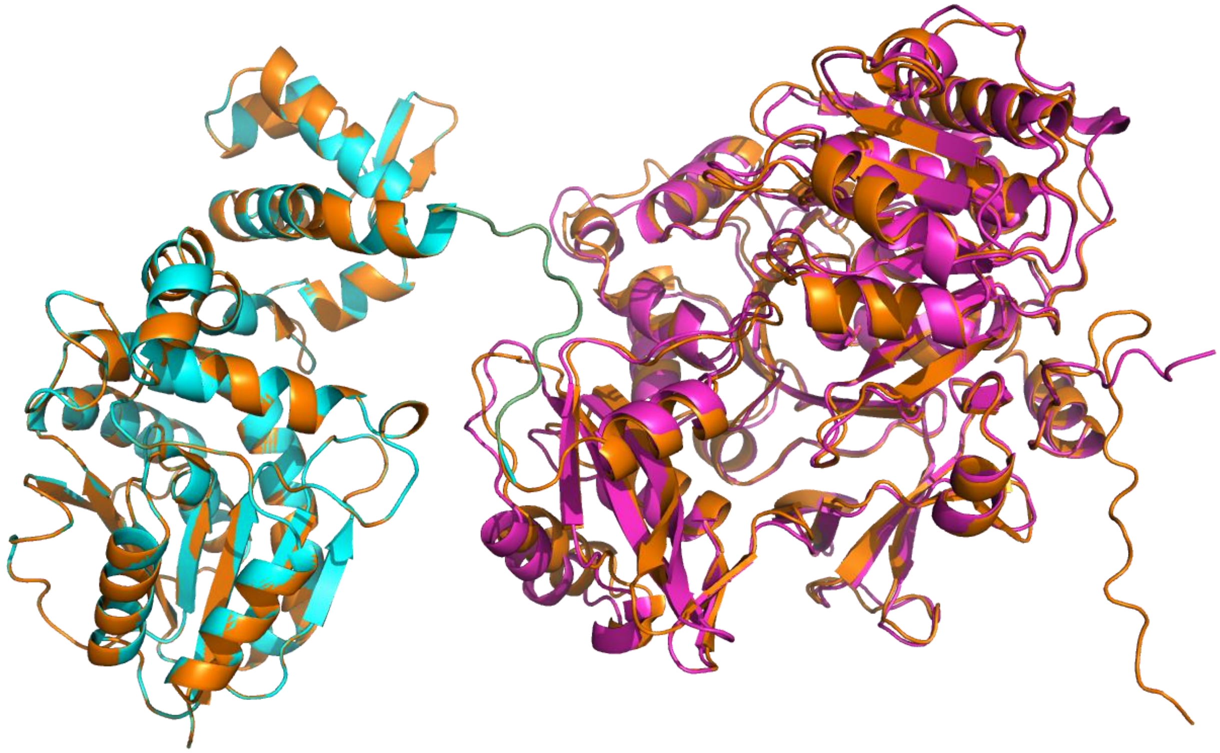

Figure S14. Superimposition of the AlphaFold-predicted structures of Dip21 (magenta) and Dip20 (cyan) to the N-terminal synthetase and C-terminal methylase domains of QbsL (orange), respectively.

Table S1. The biosynthetic gene clusters (BGCs) of secondary metabolites in *Amycolatopsis azurea* DSM 43854 analyzed from antiSMASH 5.

Cluster	Type	From	To	Most similar known BGC (% similarity)
1.1	CDPS	137,563	158,264	
1.2	terpene	210,895	233,051	geosmin (100%)
1.3	ectoine	256,222	266,611	ectoine (100%)
2.1	T1PKS	318	68,485	butyrolactol A (66%)
2.2	hglE-KS, T1PKS	108,031	158,424	rimosamide (14%)
2.3	lassopeptide	268,408	290,889	ansacarbamitocin A (12%)
2.4	NRPS, amglyccycl	324,333	378,452	acarbose (14%)
2.5	NRPS, T1PKS	378,800	487,216	caerulomycin A (52%)
2.6	NRPS	548,461	609,364	

2.7	T1PKS	610,946	657,329	kanamycin (1%)
5.1	lanthipeptide	90,029	112,572	Ery-9 / Ery-6 / Ery-8 / Ery-7 / Ery-5 / Ery-4 / Ery-3 (75%)
6.1	terpene	166,574	187,503	isorenieratene (42%)
7.1	NRPS-like	10,140	54,042	ansacarbamitocin A (4%)
10.1	NRPS	16,609	84,811	mirubactin (78%)
11.1	terpene	23,959	46,151	enduracidin (10%)
11.2	betalactone	66,497	91,707	
12.1	arylpolyene	78,681	119,832	kinamycin (5%)
15.1	NRPS	116,412	178,671	albachelin (100%)
16.1	bacteriocin	162,646	173,458	
16.2	terpene	364,878	386,005	vazabitide A (4%)
16.3	T1PKS	432,515	469,723	funisamine (22%)
20.1	other, arylpolyene	21,861	73,484	kedarcidin (19%)
22.1	NRPS-like	181,449	225,240	meilingmycin (3%)
23.1	NRPS, CDPS, T1PKS, T3PKS, terpene	1,589	122,641	diazepinomicin (17%)
24.1	terpene	65,035	86,174	2-methylisoborneol (100%)
24.2	NRPS-like, lanthipeptide	108,423	151,788	
24.3	NRPS	214,282	273,246	capreomycin IA / capreomycin IB / capreomycin IIA / capreomycin IIB (9%)
25.1	T3PKS, NRPS-like, NRPS	124,067	226,706	keratinimicin A / keratinimicin B / keratinimicin C / keratinimicin D (92%)
25.2	T1PKS, NRPS-like	233,345	279,671	polyoxypeptin (10%)
27.1	lanthipeptide	16,974	39,844	
28.1	T1PKS	3,842	49,676	amycolamycin A / amycolamycin B (31%)
28.2	T2PKS	134,047	206,532	actinorhodin (59%)
29.1	hglE-KS, T1PKS	14,179	64,527	rifamorpholine A / rifamorpholine B / rifamorpholine C / rifamorpholine D / rifamorpholine E (9%)
33.1	T1PKS, NRPS	1	46,481	chlortetracycline (5%)
45.1	T1PKS	1	26,493	

Table S2. Machine-learning predicted probability scores for various bioactivities of the AntiSMASH 5.0 annotated BGCs from *A. azurea DSM 43854*. The scores were obtained using three classifiers [extra trees, logistic regression, and support vector machine (SVM)] and were averaged for each activity classification.

	Antibacterial				Anti-gram positive				Anti-gram negative				antifungal/antitumor/cytotoxic				Antifungal				Antitumor/cytotoxic			
Cluster	Tree	Log	SVM	Avg	Tree	Log	SVM	Avg	Tree	Log	SVM	Avg	Tree	Log	SVM	Avg	Tree	Log	SVM	Avg	Tree	Log	SVM	Avg
1.1	0.68	0.50	0.56	0.58	0.56	0.27	0.28	0.37	0.24	0.05	0.04	0.11	0.36	0.38	0.33	0.36	0.19	0.13	0.05	0.12	0.26	0.28	0.30	0.28
1.2	0.35	0.37	0.60	0.44	0.48	0.28	0.35	0.37	0.12	0.15	0.19	0.15	0.28	0.19	0.18	0.22	0.20	0.23	0.18	0.20	0.10	0.14	0.12	0.12
1.3	0.46	0.35	0.17	0.33	0.40	0.15	0.17	0.24	0.00	0.09	0.09	0.06	0.20	0.23	0.22	0.22	0.07	0.16	0.09	0.11	0.03	0.06	0.05	0.05
2.1	0.36	0.28	0.42	0.35	0.44	0.11	0.20	0.25	0.12	0.15	0.17	0.15	0.80	0.89	0.92	0.87	0.42	0.54	0.34	0.43	0.44	0.25	0.44	0.38
2.2	0.66	0.53	0.62	0.60	0.70	0.46	0.51	0.56	0.60	0.35	0.23	0.39	0.80	0.69	0.75	0.75	0.46	0.36	0.30	0.37	0.38	0.12	0.23	0.24
2.3	0.90	0.86	0.98	0.91	0.78	0.98	0.92	0.89	0.48	0.36	0.27	0.37	0.44	0.10	0.10	0.21	0.16	0.12	0.26	0.18	0.21	0.04	0.20	0.15
2.4	0.72	0.75	0.72	0.73	0.62	0.39	0.51	0.51	0.52	0.92	0.43	0.62	0.72	0.96	0.93	0.87	0.51	0.45	0.25	0.40	0.64	0.04	0.35	0.35
2.5	0.60	0.99	0.71	0.76	0.60	1.00	0.95	0.85	0.40	0.91	0.42	0.58	0.72	0.92	0.93	0.86	0.62	0.27	0.17	0.35	0.50	0.07	0.35	0.31
2.6	0.64	0.52	0.59	0.58	0.48	0.35	0.32	0.38	0.20	0.10	0.11	0.14	0.40	0.82	0.65	0.62	0.34	0.19	0.17	0.23	0.21	0.60	0.32	0.38
2.7	0.66	0.53	0.62	0.60	0.64	0.26	0.41	0.44	0.24	0.29	0.35	0.29	0.45	0.03	0.06	0.18	0.37	0.18	0.16	0.24	0.40	0.04	0.31	0.25
5	0.58	0.69	0.68	0.65	0.62	0.73	0.63	0.66	0.32	0.52	0.30	0.38	0.20	0.04	0.07	0.10	0.28	0.12	0.19	0.20	0.11	0.01	0.08	0.06
6	0.20	0.23	0.09	0.17	0.20	0.04	0.10	0.11	0.28	0.10	0.17	0.18	0.24	0.14	0.28	0.22	0.18	0.16	0.28	0.21	0.13	0.04	0.06	0.08
7	0.52	0.56	0.71	0.59	0.80	0.74	0.79	0.78	0.32	0.07	0.24	0.21	0.36	0.12	0.11	0.20	0.32	0.11	0.17	0.20	0.14	0.15	0.32	0.21
10	0.46	0.27	0.66	0.46	0.38	0.00	0.05	0.14	0.36	0.00	0.03	0.13	0.48	0.01	0.02	0.17	0.28	0.05	0.16	0.16	0.45	0.00	0.33	0.26
11.1	0.78	0.78	0.70	0.75	0.80	0.93	0.79	0.84	0.16	0.07	0.09	0.11	0.40	0.13	0.17	0.23	0.22	0.13	0.17	0.17	0.32	0.11	0.21	0.22
11.2	0.70	0.54	0.82	0.69	0.54	0.84	0.80	0.73	0.32	0.09	0.10	0.17	0.48	0.44	0.37	0.43	0.10	0.15	0.20	0.15	0.30	0.69	0.42	0.47
12	0.84	0.78	0.71	0.77	0.74	0.89	0.88	0.84	0.32	0.13	0.33	0.26	0.36	0.00	0.06	0.14	0.10	0.04	0.13	0.09	0.29	0.05	0.29	0.21
15	0.26	0.43	0.57	0.42	0.36	0.36	0.42	0.38	0.28	0.22	0.34	0.28	0.36	0.14	0.13	0.21	0.22	0.06	0.16	0.15	0.15	0.03	0.29	0.15
16.1	0.52	0.39	0.43	0.45	0.24	0.31	0.28	0.28	0.00	0.08	0.09	0.05	0.00	0.21	0.22	0.14	0.01	0.17	0.18	0.12	0.02	0.14	0.06	0.07
16.2	0.71	0.50	0.40	0.54	0.52	0.34	0.36	0.41	0.20	0.15	0.19	0.18	0.12	0.14	0.10	0.12	0.11	0.06	0.06	0.08	0.08	0.20	0.08	0.12
16.3	0.62	0.48	0.40	0.50	0.60	0.50	0.56	0.55	0.04	0.11	0.17	0.11	0.72	0.32	0.24	0.42	0.34	0.32	0.21	0.29	0.45	0.07	0.13	0.22
20	0.54	0.49	0.69	0.57	0.76	0.26	0.37	0.47	0.08	0.00	0.06	0.05	0.68	0.26	0.36	0.44	0.30	0.18	0.19	0.22	0.59	0.22	0.38	0.40
22	0.82	0.87	0.70	0.80	0.78	0.97	0.87	0.87	0.44	0.75	0.45	0.55	0.52	0.35	0.47	0.45	0.24	0.12	0.18	0.18	0.44	0.04	0.30	0.26
23	0.50	0.60	0.70	0.60	0.58	0.39	0.69	0.55	0.56	0.34	0.34	0.42	0.60	0.96	0.98	0.85	0.46	0.47	0.17	0.37	0.57	0.69	0.35	0.54
24.1	0.52	0.45	0.15	0.37	0.62	0.31	0.32	0.42	0.16	0.10	0.09	0.12	0.24	0.31	0.29	0.28	0.15	0.19	0.09	0.14	0.14	0.27	0.17	0.19
24.2	0.58	0.60	0.52	0.57	0.76	0.35	0.50	0.54	0.16	0.03	0.06	0.08	0.28	0.03	0.07	0.13	0.23	0.12	0.17	0.17	0.15	0.02	0.20	0.12
24.3	0.68	0.69	0.67	0.68	0.58	0.55	0.69	0.61	0.36	0.23	0.26	0.28	0.56	0.44	0.41	0.47	0.42	0.26	0.16	0.28	0.35	0.12	0.25	0.24
25.1	0.98	0.96	0.83	0.93	1.00	0.98	0.95	0.97	0.48	0.56	0.26	0.43	0.16	0.01	0.02	0.06	0.02	0.03	0.12	0.06	0.11	0.00	0.19	0.10
25.2	0.64	0.46	0.44	0.51	0.56	0.28	0.33	0.39	0.40	0.09	0.12	0.20	0.64	0.63	0.51	0.59	0.34	0.27	0.19	0.27	0.39	0.34	0.31	0.35
27	0.82	0.66	0.97	0.82	0.80	0.96	0.93	0.90	0.08	0.02	0.04	0.05	0.44	0.26	0.30	0.33	0.16	0.23	0.17	0.19	0.34	0.28	0.38	0.34
28.1	0.76	0.65	0.65	0.69	0.64	0.41	0.60	0.55	0.16	0.07	0.12	0.12	0.68	0.20	0.23	0.37	0.08	0.04	0.12	0.08	0.41	0.55	0.40	0.45
28.2	0.72	0.77	0.70	0.73	0.54	0.87	0.81	0.74	0.44	0.01	0.11	0.19	0.68	0.83	0.84	0.78	0.36	0.12	0.18	0.22	0.63	0.64	0.35	0.54
29	0.50	0.63	0.66	0.60	0.54	0.19	0.38	0.37	0.28	0.10	0.13	0.17	0.60	0.44	0.36	0.47	0.24	0.12	0.19	0.19	0.51	0.31	0.42	0.41
33	0.78	0.69	0.64	0.70	0.62	0.69	0.60	0.64	0.24	0.63	0.48	0.45	0.72	0.57	0.57	0.62	0.18	0.16	0.16	0.17	0.31	0.83	0.36	0.50
45	0.72	0.70	0.60	0.67	0.70	0.80	0.71	0.74	0.20	0.13	0.28	0.20	0.48	0.19	0.34	0.33	0.22	0.17	0.19	0.19	0.35	0.07	0.19	0.20

Table S3. List of genes in the Dipyrimicin *dip* BGC, their proposed functions, and comparison to the *cae* and *col* BGC.

***dip* gene**	**Size (AA)[a]**	**BLAST Hit protein [origin]**	**ID/SI[b] (%)**	***cae* homolog**	**ID/SI[b] (%)**	***col* homolog**	**ID/SI[b] (%)**	**proposed function**
orf -3	123	YciI family protein [*Actinokineospora fastidiosa*]	90/95					
orf -2	425	RNA polymerase sigma factor [Lentzea roselyniae]	83/88					
orf -1	363	FAD-dependent oxidoreductase [*Amycolatopsis japonica*]	74/80					
1	201	TetR family transcriptional regulator [*Amycolatopsis alba*]	85/91	*caeI2*	33/50	*colI2*	30/58	regulator
2	135	hypothetical protein [*Amycolatopsis sp.* WAC 04169]	90/95					unknown
3	324	glycine betaine ABC transporter substrate-binding protein [*Amycolatopsis oliviviridis*]	96/97					putative transporter
4	822	ABC transporter permease subunit [*Amycolatopsis sp.* WAC 01416]	94/95					putative transporter
5	606	asparagine synthase (glutamine hydrolyzing) [*Fodinicola feengrottensis*]	62/73					putative amidotransferase
6	403	MFS transporter [*Streptomyces sp.* NPDC004629]	60/74	*caeH3*	43/60			putative transporter
7	611	ABC transporter ATP-binding protein [*Streptomyces sp.* NPDC004629]	68/80	*caeH1*	62/75	*colH1*	56/70	transporter
8	582	ABC transporter ATP-binding protein [*Streptomyces sp.* NPDC004629]	70/78	*caeH2*	63/75	*colH2*	59/71	transporter

9	369	NAD(P)/FAD-dependent oxidoreductase [*Amycolatopsis anabasis*]	65/71	*caeB6*	54/66			putative hydroxylase
10	231	alpha/beta fold hydrolase [*Streptomyces sp.* NPDC004629]	67/76					putative hydrolase
11b	542	(2,3-dihydroxybenzoyl)adenylate synthase [*Amycolatopsis anabasis*]	73/81	*caeA1*	67/75	*colA1b*	54/63	AMP ligase
11a	72	acyl carrier protein [*Micromonospora craniellae*]	66/80			*colA1a*	46/69	acyl carrier protein
12	417	DegT/DnrJ/EryC1/StrS family aminotransferase family protein [*Streptoalloteichus tenebrarius*]	75/85	*caeP1*	70/80	*colP1*	62/74	aminotransferase
13	395	FAD-dependent oxidoreductase [*Amycolatopsis anabasis*]	76/84	*caeP2*	71/81	*colP2*	59/72	oxidase
14	2475	NRPS-T1PKS [*Streptoalloteichus tenebrarius*]	72/80	*caeA2*	68/77	*colA2*	58/69	NRPS/PKS
15	1058	non-ribosomal peptide synthetase [*Amycolatopsis anabasis*]	75/83	*caeA3*	71/79	*colA3*	52/65	NRPS
16	379	acyl-CoA dehydrogenase family protein [*Amycolatopsis anabasis*]	78/88	*caeB1*	79/87	*colB1*	62/74	acyl-CoA dehydrogenase
17	251	thioesterase II family protein [*Amycolatopsis anabasis*]	63/74	*caeA4*	62/72	*colA4*	48/63	thioesterase
18	145	response regulator transcription factor [*Amycolatopsis anabasis*]	68/76	*caeI1*	51/61	*colI1*	51/62	regulator
19	400	CrmL amidohydrolase [*Actinoalloteichus sp.* WH1-2216-6]	73/83	*caeD*	73/83	*colD*	61/73	amidohydrolase
20	351	SAM-dependent methyltransferase [*Streptomyces caatingaensis*]	65/74	*caeG1*	25/42	*colG1*	26/44	putative O-methyltransferase
21	550	class I adenylate-forming enzyme family protein [*Streptomyces caatingaensis*]	64/73					putative AMP-dependent synthetase/ligase
orf 1	394	MFS transporter [*Amycolatopsis sp.* NEAU-NG30]	89/91					
orf 2	227	FadR/GntR family transcriptional regulator [*Streptomyces caatingaensis*]	66/75					
orf 3	149	MerR family transcriptional regulator [*Amycolatopsis sp.* NEAU-NG30]	83/88					

[a] denotes amino acids

[b] denotes Identity/Similarity (%)

Table S4. First 10 FoldSeek structural search hits for putative hydroxylase Dip9 from PDB and AlphaFold Databases.

Target	Database	Description	Scientific Name	Prob.	Seq. Id.	TM-score	Score
3alm	PDB100	Crystal structure of 2-methyl-3-hydroxypyridine-5-carboxylic acid oxygenase, mutant C294A	*Mesorhizobium japonicum MAFF 303099*	1	38.8	0.922	92
4h2p	PDB100	Tetrameric form of 2-methyl-3-hydroxypyridine-5-carboxylic acid oxygenase (MHPCO)	*Mesorhizobium japonicum MAFF 303099*	1	38.8	0.922	92
3all	PDB100	Crystal structure of 2-methyl-3-hydroxypyridine-5-carboxylic acid oxygenase, mutant Y270A	*Mesorhizobium japonicum MAFF 303099*	1	38.6	0.921	92

4h2n	PDB100	Crystal structure of MHPCO, Y270F mutant	*Mesorhizobium japonicum MAFF 303099*	1	38.6	0.921	92
4bk3	PDB100	Crystal structure of 3-hydroxybenzoate 6-hydroxylase uncovers lipid- assisted flavoprotein strategy for regioselective aromatic hydroxylation: Y105F mutant	*Rhodococcus jostii RHA1*	0.99	21.9	0.859	88
4bk2	PDB100	Crystal structure of 3-hydroxybenzoate 6-hydroxylase uncovers lipid- assisted flavoprotein strategy for regioselective aromatic hydroxylation: Q301E mutant	*Rhodococcus jostii RHA1*	0.99	22.1	0.858	88
4bjz	PDB100	Crystal structure of 3-hydroxybenzoate 6-hydroxylase uncovers lipid- assisted flavoprotein strategy for regioselective aromatic hydroxylation: Native data	*Rhodococcus jostii RHA1*	0.99	22.1	0.858	88
4bjy	PDB100	Crystal structure of 3-hydroxybenzoate 6-hydroxylase uncovers lipid- assisted flavoprotein strategy for regioselective aromatic hydroxylation: Platinum derivative	*Rhodococcus jostii RHA1*	0.99	21.9	0.857	88
3rp8	PDB100	Crystal Structure of Klebsiella pneumoniae R204Q HpxO complexed with FAD	*Klebsiella pneumoniae subsp. pneumoniae MGH 78578*	0.99	19.4	0.853	86
4bk1	PDB100	Crystal structure of 3-hydroxybenzoate 6-hydroxylase uncovers lipid- assisted flavoprotein strategy for regioselective aromatic hydroxylation: H213S mutant in complex with 3-hydroxybenzoate	*Rhodococcus jostii RHA1*	0.99	21.6	0.852	87
AF-A0A0E4AFG7-F1-model_v4	AFDB-SWISSPROT	Putative 2-heptyl-3-hydroxy-4(1H)-quinolone synthase AqdB1	*Rhodococcus erythropolis*	1	35.5	0.914	92
AF-B1MFK1-F1-model_v4	AFDB-SWISSPROT	2-heptyl-3-hydroxy-4(1H)-quinolone synthase	*Mycobacteroides abscessus ATCC 19977*	1	36	0.908	91
AF-A0A0E4AFH6-F1-model_v4	AFDB-SWISSPROT	Probable 2-heptyl-3-hydroxy-4(1H)-quinolone synthase AqdB2	*Rhodococcus erythropolis*	1	33.7	0.906	92
AF-O06489-F1-model_v4	AFDB-SWISSPROT	Putative oxidoreductase YetM	*Bacillus subtilis subsp. subtilis str. 168*	0.99	23.7	0.879	87
AF-H1ZZA4-F1-model_v4	AFDB-SWISSPROT	Aurachin C monooxygenase/isomerase	*Stigmatella aurantiaca*	0.99	22.6	0.869	88
AF-Q6F6Y2-F1-model_v4	AFDB-SWISSPROT	FAD-dependent urate hydroxylase	*Acinetobacter baylyi ADP1*	0.99	19.9	0.863	88
AF-Q9F131-F1-model_v4	AFDB-SWISSPROT	3-hydroxybenzoate 6-hydroxylase 1	*Pseudomonas alcaligenes*	0.99	22	0.86	88
AF-A6T923-F1-model_v4	AFDB-SWISSPROT	FAD-dependent urate hydroxylase	*Klebsiella pneumoniae subsp. pneumoniae MGH 78578*	0.99	20	0.857	87

AF-B6D1N4-F1-model_v4	AFDB-SWISSPROT	FAD-dependent urate hydroxylase	*Klebsiella pneumoniae*	0.99	20.2	0.856	87
AF-B5B0J6-F1-model_v4	AFDB-SWISSPROT	FAD-dependent urate hydroxylase	*Klebsiella oxytoca*	0.99	17.9	0.855	87
AF-A0A353BC83-F1-model_v4	AFDB50	FAD-dependent oxidoreductase	*Planctomycetaceae bacterium*	1	24.1	0.876	89
AF-A0A1K1MJG4-F1-model_v4	AFDB50	2-polyprenyl-6-methoxyphenol hydroxylase	*Luteibacter sp. UNCMF366Tsu5.1*	0.99	25.1	0.875	88
AF-A0A521KTI3-F1-model_v4	AFDB50	FAD-dependent monooxygenase	*Nitrospirae bacterium*	0.99	23	0.875	88
AF-A0A5C6BNZ2-F1-model_v4	AFDB50	FAD-dependent urate hydroxylase	*Symmachiella macrocystis*	0.99	21.3	0.873	88
AF-A0A0A3HX39-F1-model_v4	AFDB50	FAD_binding_3 domain-containing protein	*Ureibacillus sinduriensis BLB-1 = JCM 15800*	0.99	19	0.873	88
AF-A0A168HQA1-F1-model_v4	AFDB50	FAD_binding_3 domain-containing protein	*Paenibacillus glacialis*	1	19.5	0.873	89
AF-A0A7W1I6S8-F1-model_v4	AFDB50	FAD-dependent monooxygenase	*Actinomycetia bacterium*	0.99	22.3	0.872	88
AF-A0A4R6ULI2-F1-model_v4	AFDB50	2-polyprenyl-6-methoxyphenol hydroxylase-like FAD-dependent oxidoreductase	*Permianibacter aggregans*	0.99	18.6	0.872	86
AF-A0A379BP18-F1-model_v4	AFDB50	3-hydroxybenzoate 6-hydroxylase 1	*Nocardia brasiliensis*	0.99	21.6	0.872	87
AF-A0A1H7VYS7-F1-model_v4	AFDB50	2-polyprenyl-6-methoxyphenol hydroxylase	*Stigmatella aurantiaca*	0.99	22.3	0.871	88
AF-K0EM28-F1-model_v4	AFDB-PROTEOME	Monooxygenase	*Nocardia brasiliensis ATCC 700358*	0.99	25.4	0.872	86
AF-K0ETR9-F1-model_v4	AFDB-PROTEOME	FAD-binding monooxygenase	*Nocardia brasiliensis ATCC 700358*	0.99	21.8	0.871	87
AF-Q9HYR7-F1-model_v4	AFDB-PROTEOME	Probable FAD-dependent monooxygenase	*Pseudomonas aeruginosa PAO1*	0.99	19.3	0.866	88

AF-Q2FVW3-F1-model_v4	AFDB-PROTEOME	FAD_binding_3 domain-containing protein	*Staphylococcus aureus subsp. aureus NCTC 8325*	0.99	21	0.864	86
AF-K0ETS7-F1-model_v4	AFDB-PROTEOME	2-polyprenyl-6-methoxyphenol hydroxylase-like oxidoreductase	*Nocardia brasiliensis ATCC 700358*	0.99	21.3	0.861	86
AF-A0A0H3GN89-F1-model_v4	AFDB-PROTEOME	Putative flavoprotein monooxygenase	*Klebsiella pneumoniae subsp. pneumoniae HS11286*	0.99	20	0.857	87
AF-K0ELV8-F1-model_v4	AFDB-PROTEOME	FAD-binding monooxygenase	*Nocardia brasiliensis ATCC 700358*	0.99	21.5	0.847	86
AF-Q10RL6-F1-model_v4	AFDB-PROTEOME	FAD binding domain containing protein, expressed	*Oryza sativa Japonica Group*	0.99	19	0.847	88
AF-Q9FLC2-F1-model_v4	AFDB-PROTEOME	Monooxygenase 3	*Arabidopsis thaliana*	0.99	18.4	0.847	88
AF-O81816-F1-model_v4	AFDB-PROTEOME	Monooxygenase 2	*Arabidopsis thaliana*	0.99	19.6	0.847	88

Table S5. First 10 FoldSeek structural search hits for putative O-methyltransferae Dip20 from PDB and AlphaFold Databases.

**Target**	**Database**	**Description**	**Scientific Name**	**Prob.**	**Seq. Id.**	**TM-score**	**Score**
2r3s	PDB100	Crystal structure of a putative O-methyltransferase at 2.15 A resolution	*Nostoc punctiforme PCC 73102*	0.99	26.1	0.907	87
7clf	PDB100	PigF with SAH	*Serratia marcescens*	0.99	21	0.853	83
4a6d	PDB100	Crystal structure of human N-acetylserotonin methyltransferase (ASMT) in complex with SAM	*Homo sapiens*	0.99	22.1	0.848	84
6c5b	PDB100	Crystal Structure Analysis of LaPhzM	*Lysobacter antibioticus*	0.99	18.7	0.84	81
7wdq	PDB100	DsyB in complex with SAM	*Nisaea denitrificans DSM 18348*	0.99	22.7	0.835	81
8tjj	PDB100	SAM-dependent methyltransferase RedM bound to SAM	*uncultured bacterium*	0.98	24.6	0.831	79
5icc	PDB100	Crystal structure of (S)-norcoclaurine 6-O-methyltransferase with S-adenosyl-L-homocysteine	*Thalictrum flavum subsp. glaucum*	0.99	16.8	0.829	83
7v6l	PDB100	LcCOMT in complex with SAH	Ligusticum chuanxiong	0.99	16.8	0.827	81
3gwz	PDB100	Structure of the Mitomycin 7-O-methyltransferase MmcR	Streptomyces lavendulae	0.99	22.7	0.827	81
6yjw	PDB100	Structure of Fragaria ananassa O-methyltransferase crystallized with PAS polypeptide	Fragaria x ananassa	0.99	17.7	0.824	82
AF-A0A7Y5HBN6-F1-model_v4	AFDB50	Methyltransferase domain-containing protein	*Candidatus Brocadiae bacterium*	1	34.6	0.922	90

AF-A0A1E4G6R2-F1-model_v4	AFDB50	Uncharacterized protein	*bacterium SCN 62-11*	0.99	26.5	0.872	84
AF-A0A2S6QMZ9-F1-model_v4	AFDB50	3-hydroxy-5-methyl-1-naphthoate 3-O-methyltransferase	*Alphaproteobacteria bacterium MarineAlpha9_Bin3*	0.99	18.9	0.866	85
AF-A0A5J6GJ07-F1-model_v4	AFDB50	Methyltransf_2 domain-containing protein	*Streptomyces kanamyceticus*	0.99	22.2	0.863	83
AF-A0A2R5HC89-F1-model_v4	AFDB50	O-methyltransferase	*Mycobacterium montefiorense*	0.99	20.9	0.857	83
AF-A0A254QRY0-F1-model_v4	AFDB50	Methyltransferase	*Phaeobacter sp. 22II1-1F12B*	0.99	20.9	0.854	83
AF-A0A7C2P238-F1-model_v4	AFDB50	Methyltransferase domain-containing protein	*Schlesneria paludicola*	0.99	20.9	0.852	84
AF-A0A1H1GZC4-F1-model_v4	AFDB50	Dimerisation domain-containing protein	*Actinopolyspora saharensis*	0.99	20.6	0.851	84
AF-A0A501PB90-F1-model_v4	AFDB50	Methyltransferase	*Emcibacter nanhaiensis*	0.99	14.2	0.849	82
AF-A0A7W8JHX0-F1-model_v4	AFDB50	Putative transcriptional regulator	*Rhodanobacter sp. ANJX3*	0.99	17.9	0.849	83
AF-Q92056-F1-model_v4	AFDB-SWISSPROT	Acetylserotonin O-methyltransferase	*Gallus gallus*	0.99	20.9	0.86	85
AF-P10950-F1-model_v4	AFDB-SWISSPROT	Acetylserotonin O-methyltransferase	*Bos taurus*	0.99	21.4	0.853	84
AF-Q8HZJ0-F1-model_v4	AFDB-SWISSPROT	Acetylserotonin O-methyltransferase	*Macaca mulatta*	0.99	20.9	0.853	84
AF-P46597-F1-model_v4	AFDB-SWISSPROT	Acetylserotonin O-methyltransferase	*Homo sapiens*	0.99	21.7	0.848	84
AF-A0A0N9HTA1-F1-model_v4	AFDB-SWISSPROT	Desmethyl-yatein O-methyltransferase	*Sinopodophyllum hexandrum*	0.99	18.1	0.844	84
AF-P39896-F1-model_v4	AFDB-SWISSPROT	Tetracenomycin polyketide synthesis 8-O-methyl transferase TcmO	*Streptomyces glaucescens*	0.99	23.4	0.844	83
AF-Q42654-F1-model_v4	AFDB-SWISSPROT	Flavonoid 3'-O-methyltransferase FOMT	*Chrysosplenium americanum*	0.99	15.8	0.838	82
AF-Q54B60-F1-model_v4	AFDB-SWISSPROT	Probable inactive O-methyltransferase 11	*Dictyostelium discoideum*	0.99	14	0.838	81

AF-Q42653-F1-model_v4	AFDB-SWISSPROT	Flavone 3'-O-methyltransferase OMT2	*Chrysosplenium americanum*	0.99	16.9	0.83	82
AF-Q6WUC1-F1-model_v4	AFDB-SWISSPROT	(RS)-norcoclaurine 6-O-methyltransferase	*Papaver somniferum*	0.99	14.6	0.828	82
AF-P46597-F1-model_v4	AFDB-PROTEOME	Acetylserotonin O-methyltransferase	*Homo sapiens*	0.99	21.7	0.848	84
AF-K0F7F0-F1-model_v4	AFDB-PROTEOME	O-methyltransferase	*Nocardia brasiliensis ATCC 700358*	0.99	21.3	0.847	85
AF-A0A0R4ITX8-F1-model_v4	AFDB-PROTEOME	Acetylserotonin O-methyltransferase	*Danio rerio*	0.99	20.9	0.846	83
AF-K0ESM0-F1-model_v4	AFDB-PROTEOME	O-demethylpuromycin-O-methyltransferase	*Nocardia brasiliensis ATCC 700358*	0.99	21.2	0.839	84
AF-A3KNM1-F1-model_v4	AFDB-PROTEOME	Acetylserotonin O-methyltransferase 2	*Danio rerio*	0.99	20.8	0.838	83
AF-Q54B60-F1-model_v4	AFDB-PROTEOME	Probable inactive O-methyltransferase 11	*Dictyostelium discoideum*	0.99	14	0.838	81
AF-K0EN01-F1-model_v4	AFDB-PROTEOME	O-methyltransferase family protein	*Nocardia brasiliensis ATCC 700358*	0.99	26.2	0.834	82
AF-Q86I40-F1-model_v4	AFDB-PROTEOME	O-methyltransferase 4	*Dictyostelium discoideum*	0.99	12.8	0.83	81
AF-O53764-F1-model_v4	AFDB-PROTEOME	Probable methyltransferase/methylase	*Mycobacterium tuberculosis H37Rv*	0.99	21.9	0.829	81
AF-A0A0R4J3I5-F1-model_v4	AFDB-PROTEOME	Uncharacterized protein	*Glycine max*	0.99	17.7	0.822	83

Table S6. First 10 FoldSeek structural search hits for putative amidotransferase Dip5 from PDB and AlphaFold Databases.

Target	Database	Description	Scientific Name	Prob.	Seq. Id.	TM-score	Score
7ylz	PDB100	Unliganded form of hydroxyamidotransferase TsnB9	*Streptomyces sp. RM72*	1	37.5	0.937	91
1ct9	PDB100	crystal structure of asparagine synthetase B from Escherichia coli	*Escherichia coli*	0.96	20.5	0.818	73
6gq3	PDB100	Human asparagine synthetase (ASNS) in complex with 6-diazo-5-oxo-L-norleucine (DON) at 1.85 A resolution	*Homo sapiens*	0.96	20.5	0.798	73
1mc1	PDB100	beta-lactam synthetase with product (DGPC), AMP and PPI	*Streptomyces clavuligerus*	0.92	16.3	0.759	69
1mbz	PDB100	beta-lactam synthetase with trapped intermediate	*Streptomyces clavuligerus*	0.91	16.3	0.758	68
1mb9	PDB100	beta-lactam synthetase complexed with ATP	*Streptomyces clavuligerus*	0.91	16.3	0.757	68

1jgt	PDB100	crystal structure of beta-lactam synthetase	*Streptomyces clavuligerus*	0.91	16.4	0.754	68
1m1z	PDB100	beta-lactam synthetase apo enzyme	*Streptomyces clavuligerus*	0.9	16.4	0.753	67
1q19	PDB100	Carbapenam Synthetase	*Pectobacterium carotovorum*	0.91	11.7	0.746	68
1q15	PDB100	Carbapenam Synthetase	*Pectobacterium carotovorum*	0.89	11.5	0.738	66
AF-Q9I231-F1-model_v4	AFDB-PROTEOME	Probable asparagine synthetase	*Pseudomonas aeruginosa PAO1*	1	45	0.971	97
AF-K0EYT1-F1-model_v4	AFDB-PROTEOME	Putative amidotransferase	*Nocardia brasiliensis ATCC 700358*	1	45.8	0.964	97
AF-Q9I781-F1-model_v4	AFDB-PROTEOME	Potential phenazine-modifying enzyme	*Pseudomonas aeruginosa PAO1*	1	40.7	0.96	96
AF-K0FD31-F1-model_v4	AFDB-PROTEOME	Asparagine synthetase	*Nocardia brasiliensis ATCC 700358*	1	43.3	0.956	96
AF-Q86A01-F1-model_v4	AFDB-PROTEOME	Asparagine synthetase	*Dictyostelium discoideum*	1	32.8	0.934	96
AF-Q9HYE7-F1-model_v4	AFDB-PROTEOME	Probable glutamine amidotransferase	*Pseudomonas aeruginosa PAO1*	1	29	0.902	89
AF-A0A132Z0T1-F1-model_v4	AFDB-PROTEOME	Asparagine synthase (Glutamine-hydrolyzing)	*Enterococcus faecium*	0.99	24	0.867	88
AF-P9WN33-F1-model_v4	AFDB-PROTEOME	Putative asparagine synthetase [glutamine-hydrolyzing]	*Mycobacterium tuberculosis H37Rv*	0.99	24.1	0.858	88
AF-K0EXR4-F1-model_v4	AFDB-PROTEOME	Asparagine synthase	*Nocardia brasiliensis ATCC 700358*	0.99	22.1	0.85	88
AF-Q9CCF2-F1-model_v4	AFDB-PROTEOME	Putative asparagine synthetase	*Mycobacterium leprae TN*	0.99	23.2	0.845	88
AF-A0A0K0QAS6-F1-model_v4	AFDB50	Asparagine synthase	*Bacillus thuringiensis*	1	41.8	0.971	97
AF-A0A0B5NI93-F1-model_v4	AFDB50	Asparagine synthase (Glutamine-hydrolyzing)	*Bacillus thuringiensis*	1	42.2	0.971	97
AF-A0A854KS77-F1-model_v4	AFDB50	Asparagine synthase (Glutamine-hydrolyzing)	*Bacillus thuringiensis serovar shandongiensis*	1	42.4	0.971	97
AF-A0A090YNR9-F1-model_v4	AFDB50	Asparagine synthase	*Bacillus clarus*	1	42.1	0.971	97
AF-A0A373KWD5-F1-model_v4	AFDB50	Asparagine synthase (Glutamine-hydrolyzing)	*Roseburia sp. AF42-8*	1	36.2	0.969	97
AF-R5JEM6-F1-model_v4	AFDB50	Asparagine synthetase	*Coprococcus sp. CAG:782*	1	33.3	0.968	97

AF-R7RSB4-F1-model_v4	AFDB50	Asparagine synthetase [glutamine-hydrolyzing]	*Thermobrachium celere DSM 8682*	1	34.9	0.968	97
AF-A0A6G2UGB8-F1-model_v4	AFDB50	Asparagine synthase (Glutamine-hydrolyzing)	*Streptomyces sp. SID4931*	1	46.4	0.967	97
AF-A0A3M8H280-F1-model_v4	AFDB50	Asparagine synthase (Glutamine-hydrolyzing)	*Lysinibacillus halotolerans*	1	39.6	0.967	97
AF-A0A7K0CDT8-F1-model_v4	AFDB50	Asparagine synthetase [glutamine-hydrolyzing] 3	*Streptomyces smaragdinus*	1	45.5	0.962	97
AF-O05272-F1-model_v4	AFDB-SWISSPROT	Asparagine synthetase [glutamine-hydrolyzing] 3	*Bacillus subtilis subsp. subtilis str. 168*	1	40.7	0.968	97
AF-P54420-F1-model_v4	AFDB-SWISSPROT	Asparagine synthetase [glutamine-hydrolyzing] 1	*Bacillus subtilis subsp. subtilis str. 168*	0.99	25.8	0.872	88
AF-P9WN33-F1-model_v4	AFDB-SWISSPROT	Putative asparagine synthetase [glutamine-hydrolyzing]	*Mycobacterium tuberculosis H37Rv*	0.99	24.1	0.858	88
AF-P64248-F1-model_v4	AFDB-SWISSPROT	Putative asparagine synthetase [glutamine-hydrolyzing]	*Mycobacterium tuberculosis variant bovis AF2122/97*	0.99	24.1	0.858	88
AF-P9WN32-F1-model_v4	AFDB-SWISSPROT	Putative asparagine synthetase [glutamine-hydrolyzing]	*Mycobacterium tuberculosis CDC1551*	0.99	24	0.858	88
AF-B6HLP8-F1-model_v4	AFDB-SWISSPROT	Amidase chyE	*Penicillium rubens Wisconsin 54-1255*	1	19.4	0.855	89
AF-I1S3K8-F1-model_v4	AFDB-SWISSPROT	Amidase chry2	*Fusarium graminearum PH-1*	1	19.6	0.855	89
AF-O24338-F1-model_v4	AFDB-SWISSPROT	Asparagine synthetase [glutamine-hydrolyzing]	*Sandersonia aurantiaca*	0.96	21.5	0.804	74
AF-Q54MB4-F1-model_v4	AFDB-SWISSPROT	Probable asparagine synthetase [glutamine-hydrolyzing]	*Dictyostelium discoideum*	0.97	20.8	0.785	75
AF-P22106-F1-model_v4	AFDB-SWISSPROT	Asparagine synthetase B [glutamine-hydrolyzing]	*Escherichia coli K-12*	0.96	21.1	0.781	74

Table S7. First 10 FoldSeek structural search hits for putative synthetase/ligase Dip21 from PDB and AlphaFold Databases.

Target	Database	Description	Scientific Name	Prob.	Seq. Id.	TM-score	Score
1mdf	PDB100	Crystal structure of DhbE in absence of substrate	*Bacillus subtilis*	1	28.7	0.913	90
7tz4	PDB100	Salicylate Adenylate PchD from Pseudomonas aeruginosa containing 4-cyanosalicyl-AMS	*Pseudomonas aeruginosa*	0.99	28.8	0.899	88
7tyb	PDB100	Salicylate Adenylate PchD from Pseudomonas aeruginosa containing salicyl-AMS	*Pseudomonas aeruginosa PAO1*	0.99	28.8	0.895	87
7kyd	PDB100	Drosophila melanogaster long-chain fatty-acyl-CoA synthetase	*Drosophila melanogaster*	0.99	20.4	0.869	85

		CG6178					
4gxr	PDB100	Structure of ATP bound RpMatB-BxBclM chimera B3	*Rhodopseudomonas palustris CGA009*	0.99	22.5	0.869	83
2v7b	PDB100	Crystal structures of a benzoate CoA ligase from Burkholderia xenovorans LB400	*Paraburkholderia xenovorans LB400*	0.99	20.2	0.867	81
4gxq	PDB100	Crystal Structure of ATP bound RpMatB-BxBclM chimera B1	*Rhodopseudomonas palustris CGA009*	0.99	23.2	0.866	83
4wv3	PDB100	Crystal structure of the anthranilate CoA ligase AuaEII in complex with anthranoyl-AMP	*Stigmatella aurantiaca*	0.99	21.3	0.866	84
5bsw	PDB100	Crystal structure of 4-coumarate:CoA ligase delta-V341 mutant complexed with feruloyl adenylate	*Nicotiana tabacum*	0.99	21.3	0.862	84
6h1b	PDB100	Structure of amide bond synthetase Mcba K483A mutant from Marinactinospora thermotolerans	*Marinactinospora thermotolerans*	0.99	28.8	0.861	81
AF-A0A534XEG6-F1-model_v4	AFDB50	Cyclohexanecarboxylate-CoA ligase	*Deltaproteobacteria bacterium*	1	30.4	0.924	92
AF-A0A7Y0JBY0-F1-model_v4	AFDB50	Acyl--CoA ligase	*Streptomyces sp. GMY02*	1	39.2	0.918	90
AF-A0A6J7NLY6-F1-model_v4	AFDB50	Unannotated protein	*freshwater metagenome*	1	26.3	0.905	90
AF-A0A1C5LZ60-F1-model_v4	AFDB50	Short-chain-fatty-acid--CoA ligase	*uncultured Clostridium sp.*	1	24.8	0.902	90
AF-A0A1I7GUI0-F1-model_v4	AFDB50	Cyclohexanecarboxylate-CoA ligase	*Alicyclobacillus macrosporangiidus*	1	28.2	0.896	89
AF-A0A7C2Q990-F1-model_v4	AFDB50	Long-chain fatty acid--CoA ligase	*Armatimonadetes bacterium*	0.99	29.2	0.893	86
AF-A0A534X5V8-F1-model_v4	AFDB50	Cyclohexanecarboxylate-CoA ligase	*Deltaproteobacteria bacterium*	0.99	31	0.892	86
AF-A0A6J7NHN7-F1-model_v4	AFDB50	Unannotated protein	*freshwater metagenome*	0.99	23.3	0.888	88
AF-A0A7G3AAM8-F1-model_v4	AFDB50	Putative acyl-coa synthetase family member 2 isoform x2	*Lutzomyia longipalpis*	0.99	23.3	0.886	88
AF-A0A235HXW3-F1-model_v4	AFDB50	Long-chain fatty acid--CoA ligase	*Nostoc sp. 'Peltigera membranacea cyanobiont' 210A*	0.99	25.6	0.883	84

AF-K0EMM1-F1-model_v4	AFDB-PROTEOME	Hydroxybenzoate-AMP ligase	*Nocardia brasiliensis ATCC 700358*	1	28.7	0.919	90
AF-K0ER74-F1-model_v4	AFDB-PROTEOME	(2,3-dihydroxybenzoyl)adenylate synthase	*Nocardia brasiliensis ATCC 700358*	1	27.3	0.895	89
AF-K0F5E8-F1-model_v4	AFDB-PROTEOME	Uncharacterized protein	*Nocardia brasiliensis ATCC 700358*	0.99	28	0.882	85
AF-I6Y0X0-F1-model_v4	AFDB-PROTEOME	Probable fatty-acid-CoA ligase FadD35 (Fatty-acid-CoA synthetase) (Fatty-acid-CoA synthase)	*Mycobacterium tuberculosis H37Rv*	0.99	28.2	0.88	87
AF-P31552-F1-model_v4	AFDB-PROTEOME	Crotonobetaine/carnitine--CoA ligase	*Escherichia coli K-12*	0.99	19.1	0.874	84
AF-Q9I0S7-F1-model_v4	AFDB-PROTEOME	Probable AMP-binding enzyme	*Pseudomonas aeruginosa PAO1*	0.99	26	0.872	88
AF-Q8ZRX4-F1-model_v4	AFDB-PROTEOME	Crotonobetaine/carnitine--CoA ligase	*Salmonella enterica subsp. enterica serovar Typhimurium str. LT2*	0.99	19.3	0.872	84
AF-C1GZT6-F1-model_v4	AFDB-PROTEOME	Short-chain-fatty-acid-CoA ligase	*Paracoccidioides lutzii Pb01*	0.99	22	0.871	88
AF-Q9VDU4-F1-model_v4	AFDB-PROTEOME	Uncharacterized protein	*Drosophila melanogaster*	0.99	15.7	0.87	86
AF-Q5F969-F1-model_v4	AFDB-PROTEOME	Long-chain fatty acid--CoA ligase	*Neisseria gonorrhoeae FA 1090*	0.99	21.9	0.867	84
AF-Q84HC5-F1-model_v4	AFDB-SWISSPROT	2-hydroxy-7-methoxy-5-methyl-1-naphthoate--CoA ligase	*Streptomyces carzinostaticus*	1	28.7	0.91	91
AF-P40871-F1-model_v4	AFDB-SWISSPROT	2,3-dihydroxybenzoate-AMP ligase	*Bacillus subtilis subsp. subtilis str. 168*	1	28.2	0.909	89
AF-Q7X279-F1-model_v4	AFDB-SWISSPROT	Salicylyl-CoA synthase / salicylate adenylyltransferase	*Streptomyces sp.*	1	26.5	0.898	90
AF-Q0TLV2-F1-model_v4	AFDB-SWISSPROT	Crotonobetaine/carnitine--CoA ligase	*Escherichia coli 536*	0.99	20.2	0.888	86
AF-B4TWR4-F1-model_v4	AFDB-SWISSPROT	Crotonobetaine/carnitine--CoA ligase	*Salmonella enterica subsp. enterica serovar Schwarzengrund str. CVM19633*	0.99	20.8	0.885	85
AF-B5F750-F1-model_v4	AFDB-SWISSPROT	Crotonobetaine/carnitine--CoA ligase	*Salmonella enterica subsp. enterica serovar Agona str. SL483*	0.99	20.6	0.885	85
AF-C0Q4L3-F1-model_v4	AFDB-SWISSPROT	Crotonobetaine/carnitine--CoA ligase	*Salmonella enterica subsp. enterica serovar Paratyphi C str. RKS4594*	0.99	20.4	0.884	85
AF-B5R1R0-F1-model_v4	AFDB-SWISSPROT	Crotonobetaine/carnitine--CoA ligase	*Salmonella enterica subsp. enterica serovar Enteritidis str. P125109*	0.99	20.6	0.884	85
AF-O07610-F1-model_v4	AFDB-SWISSPROT	Long-chain-fatty-acid--CoA ligase	*Bacillus subtilis subsp. subtilis str. 168*	0.99	23.3	0.882	85
AF-O31826-F1-model_v4	AFDB-SWISSPROT	Putative acyl-CoA synthetase YngI	*Bacillus subtilis subsp. subtilis str. 168*	0.99	23.5	0.882	88

Table S8. First 10 FoldSeek structural search hits for putative hydrolase Dip10 from PDB and AlphaFold Databases.

Target	Database	Description	Scientific Name	Prob.	Seq. Id.	TM-score	Score
4l0c	PDB100	Crystal structure of the N-Fopmylmaleamic acid deformylase Nfo(S94A) from Pseudomonas putida S16	*Pseudomonas putida S16*	0.99	19.7	0.817	84
3kxp	PDB100	Crystal Structure of E-2-(Acetamidomethylene)succinate Hydrolase	*Mesorhizobium loti*	0.99	19.6	0.805	85
1zoi	PDB100	Crystal Structure of a Stereoselective Esterase from Pseudomonas putida IFO12996	*Pseudomonas putida*	0.99	15.7	0.794	85
1a88	PDB100	CHLOROPEROXIDASE L	*Streptomyces lividans*	0.99	15.3	0.793	85
5h3h	PDB100	Esterase (EaEST) from Exiguobacterium antarcticum	*Exiguobacterium antarcticum B7*	0.99	14.2	0.791	84
3hea	PDB100	The L29P/L124I mutation of Pseudomonas fluorescens esterase	*Pseudomonas fluorescens*	0.99	13.6	0.791	84
3hi4	PDB100	Switching catalysis from hydrolysis to perhydrolysis in P. fluorescens esterase	*Pseudomonas fluorescens*	0.99	13.6	0.79	84
3ia2	PDB100	Pseudomonas fluorescens esterase complexed to the R-enantiomer of a sulfonate transition state analog	*Pseudomonas fluorescens*	0.99	13.3	0.79	84
3t4u	PDB100	L29I Mutation in an Aryl Esterase from Pseudomonas fluorescens Leads to Unique Peptide Flip and Increased Activity	*Pseudomonas fluorescens*	0.99	13.6	0.79	84
4dgq	PDB100	Crystal structure of Non-heme chloroperoxidase from Burkholderia cenocepacia	*Burkholderia cenocepacia J2315*	0.99	14.2	0.787	85
AF-M2QRQ5-F1-model_v4	AFDB50	Putative hydrolase	*Amycolatopsis azurea DSM 43854*	1	99.5	0.949	91
AF-A0A7K0DV75-F1-model_v4	AFDB50	2-succinyl-6-hydroxy-2, 4-cyclohexadiene-1-carboxylate synthase	*Nocardia aurantia*	1	46.3	0.947	94
AF-A0A7H1Q9C4-F1-model_v4	AFDB50	Alpha/beta hydrolase	*Streptomyces griseofuscus*	1	40	0.946	94
AF-A0A426R654-F1-model_v4	AFDB50	Alpha/beta fold hydrolase	*Rhodococcus sp. Eu-32*	1	46.9	0.946	94
AF-A0A3A4AC57-F1-model_v4	AFDB50	Alpha/beta fold hydrolase	*Bailinhaonella thermotolerans*	1	43.1	0.938	94
AF-A0A6M4PRH7-F1-model_v4	AFDB50	Alpha/beta hydrolase	*Streptomyces sp. Jing01*	1	42.6	0.935	93
AF-A0A1G8TD96-F1-	AFDB50	Alpha/beta hydrolase family protein	*Streptomyces indicus*	1	37.7	0.934	93

model_v4							
AF-A0A387BNR8-F1-model_v4	AFDB50	Alpha/beta hydrolase	*Gryllotalpicola protaetiae*	1	41.3	0.93	94
AF-A0A652KQZ7-F1-model_v4	AFDB50	Alpha/beta fold hydrolase	*Streptomyces sp. adm13(2018)*	1	40.2	0.93	93
AF-A0A6L9Y0K9-F1-model_v4	AFDB50	Alpha/beta hydrolase	*Diaminobutyricibacter tongyongensis*	1	41.4	0.929	93
AF-Q88FY3-F1-model_v4	AFDB-SWISSPROT	N-formylmaleamate deformylase	*Pseudomonas putida KT2440*	0.99	17	0.812	86
AF-Q609V0-F1-model_v4	AFDB-SWISSPROT	Pimeloyl-[acyl-carrier protein] methyl ester esterase	*Methylococcus capsulatus str. Bath*	0.99	16	0.805	83
AF-B5FFE9-F1-model_v4	AFDB-SWISSPROT	Pimeloyl-[acyl-carrier protein] methyl ester esterase	*Aliivibrio fischeri MJ11*	0.99	13.8	0.8	83
AF-Q5E8N3-F1-model_v4	AFDB-SWISSPROT	Pimeloyl-[acyl-carrier protein] methyl ester esterase	*Aliivibrio fischeri ES114*	0.99	13.5	0.799	83
AF-A7MST3-F1-model_v4	AFDB-SWISSPROT	Pimeloyl-[acyl-carrier protein] methyl ester esterase	*Vibrio campbellii CAIM 519 = NBRC 15631 = ATCC 25920*	0.99	15.6	0.798	83
AF-A0KF11-F1-model_v4	AFDB-SWISSPROT	Pimeloyl-[acyl-carrier protein] methyl ester esterase	*Aeromonas hydrophila subsp. hydrophila ATCC 7966*	0.99	19.3	0.797	83
AF-O32234-F1-model_v4	AFDB-SWISSPROT	AB hydrolase superfamily protein YvaM	*Bacillus subtilis subsp. subtilis str. 168*	0.99	13.2	0.797	83
AF-A4ST17-F1-model_v4	AFDB-SWISSPROT	Pimeloyl-[acyl-carrier protein] methyl ester esterase	*Aeromonas salmonicida subsp. salmonicida A449*	0.99	17.3	0.796	82
AF-O05691-F1-model_v4	AFDB-SWISSPROT	Non-heme haloperoxidase	*Rhodococcus erythropolis*	0.99	16.5	0.796	85
AF-Q5QZC0-F1-model_v4	AFDB-SWISSPROT	Pimeloyl-[acyl-carrier protein] methyl ester esterase	*Idiomarina loihiensis L2TR*	0.99	14.3	0.795	83
AF-K0F1F1-F1-model_v4	AFDB-PROTEOME	Hydrolase	*Nocardia brasiliensis ATCC 700358*	0.99	15.5	0.797	83
AF-Q9I0C5-F1-model_v4	AFDB-PROTEOME	Chloroperoxidase	*Pseudomonas aeruginosa PAO1*	0.99	16.4	0.792	85
AF-K0ELX9-F1-model_v4	AFDB-PROTEOME	Hydrolase	*Nocardia brasiliensis ATCC 700358*	0.99	19.4	0.792	81
AF-Q8ZLI9-F1-model_v4	AFDB-PROTEOME	Pimeloyl-[acyl-carrier protein] methyl ester esterase	*Salmonella enterica subsp. enterica serovar Typhimurium str. LT2*	0.99	18.1	0.792	82
AF-A0A0H3GV49-F1-model_v4	AFDB-PROTEOME	Putative non-heme chloroperoxidase	*Klebsiella pneumoniae subsp. pneumoniae HS11286*	0.99	16.3	0.791	85
AF-A0A0D2GU4	AFDB-PROTEOM	Unplaced genomic scaffold supercont1.2, whole genome	*Fonsecaea pedrosoi CBS 271.37*	0.99	15.2	0.789	85

1-F1-model_v4	E	shotgun sequence					
AF-Q32AM6-F1-model_v4	AFDB-PROTEOME	Pimeloyl-[acyl-carrier protein] methyl ester esterase	*Shigella dysenteriae Sd197*	0.99	14.6	0.788	82
AF-P13001-F1-model_v4	AFDB-PROTEOME	Pimeloyl-[acyl-carrier protein] methyl ester esterase	*Escherichia coli K-12*	0.99	14.6	0.787	82
AF-I6YGL1-F1-model_v4	AFDB-PROTEOME	Possible hydrolase	*Mycobacterium tuberculosis H37Rv*	0.99	16.9	0.785	82
AF-X8FLZ2-F1-model_v4	AFDB-PROTEOME	Alpha/beta hydrolase fold family protein	*Mycobacterium ulcerans str. Harvey*	0.99	17.5	0.783	86

Table S9. Antibacterial activities of dipyrimicin A and B reported as minimum inhibitory activity.

	Antibacterial activity (µg/mL)		
	Dipyrimicin A **(1)**	Dipyrimicin B **(2)**	Tetracycline (control)
Bacillus spizizenii ATCC 6633	32	>128	0.25
Escherichia coli MG1655 ATCC 700926	128	>128	2
Pseudomonas aeruginosa ATCC 27853	>128	NT[a]	8
Staphylococcus aureus ATCC 25923	64	NT[a]	0.25

[a]NT = not tested.

Table S10. ML-predicted antibacterial activity probability of BGCs encoding 2,2'-BP genes described in **Figure 6**.

**Strain**	**NCBI RefSeq_Assembly**	***cae* % similarity[a]**	***col* % similarity[a]**	**Average antibacterial activity score**
Actinosynnema sp. ALI-1.44	GCF_001984155.1_ASM198415v1_genomic	52%	59%	74%
Streptomyces caatingaensis CMAA 1322	GCF_001187435.1_ASM118743v1_genomic	48%	38%	74%
Amycolatopsis anabasis EGI 650086	GCF_009765355.1_ASM976535v1_genomic	80%	59%	74%
Nocardia panacis YIM PH 21724	GCF_003598715.1_ASM359871v1_genomic	72%	55%	73%
Kitasatospora albolonga YIM 101047	GCF_002082585.1_ASM208258v1_genomic	68%	88%	70%
Streptomyces sp. SID14515	GCF_010548505.1_ASM1054850v1_genomic	60%	66%	67%
Streptomyces sp. Ncost-T6T-1	GCF_002705685.1_ASM270568v1_genomic	60%	77%	63%
Streptomyces filamentosus NRRL 15998	GCF_000156455.1_ASM15645v1_genomic	56%	70%	60%
Micromonospora craniellae LHW63014	GCF_014764405.1_ASM1476440v1_genomic	60%	62%	59%
Streptomyces filamentosus NRRL 11379	GCF_000156695.2_ASM15669v2_genomic	60%	74%	59%
Streptomyces sp. Cmuel-A718b	GCF_900092005.1_IMG-	60%	74%	56%

	taxon_2657245729_annotated_assembly_genomic			
Micromonospora tulbaghiae CNY-010	GCF_003612775.1_ASM361277v1_genomic	56%	59%	54%
Streptomyces sp. CS149	GCF_003024215.1_ASM302421v1_genomic	60%	70%	54%
Streptomyces sp. CAI 127	GCF_013363655.1_ASM1336365v1_genomic	60%	74%	52%
Streptomyces sp. TSRI0261	GCF_001905485.1_ASM190548v1_genomic	60%	74%	51%
Streptomyces sp. CB00271	GCF_014495705.1_ASM1449570v1_genomic	60%	74%	50%
Streptomyces sp. WA6-1-16	GCF_020341595.1_ASM2034159v1_genomic	60%	74%	49%
Streptomyces sp. CB02613	GCF_002803175.1_ASM280317v1_genomic	60%	74%	48%

[a]from AntiSMASH 5 KnownClusterBlast

References

(1) Suemori, A. Conserved and Non-Conserved Residues and Their Role in the Structure and Function of P-Hydroxybenzoate Hydroxylase. *Protein Eng Des Sel* **2013**, *26* (7), 479–488.
